# Coupling electrochemical and spectroscopic methods for river water dissolved organic matter characterization

**DOI:** 10.1007/s10661-025-14489-2

**Published:** 2025-09-01

**Authors:** Stefan Platikanov, Abel Palomas, Michelle Cedeño Mata, Romà Tauler, Jorge Villar, Ramon Bragós, Sandra Bermejo, Joaquim Jaumot, Sílvia Lacorte

**Affiliations:** 1 Department of Environmental Chemistry, IDAEA-CSIC, Jordi Girona 18-26, 08034 Barcelona, Spain; 2 Electronic Engineering Department, Polytechnic University of Catalonia (UPC), MNT Group, Jordi Girona 1-3, 08034 Barcelona, Spain; 3 Mathematics Department, Polytechnic University of Catalonia (UPC), Jordi Girona 1-3, 08034 Barcelona, Spain

**Keywords:** Electrochemical impedance spectroscopy, UV–vis, Fluorescence, Multivariate curve resolution, Data integration

## Abstract

Monitoring dissolved organic matter (DOM) in surface waters is essential for assessing ecosystem health and detecting pollution. However, conventional spectroscopic techniques often provide limited information about the electrochemical behavior of DOM. This study integrates electrochemical impedance spectroscopy (EIS) with classical methods such as UV–Vis absorption and fluorescence spectroscopies to improve DOM characterization in river water samples. In particular, this coupling provides additional insights into the electrochemical properties of DOM, which are not captured by conventional spectroscopic techniques. This study combined multiple data sources, including physicochemical parameters (e.g., water temperature, pH, conductivity), EIS spectral scores, fluorescence indices, and DOM fractions resolved by multivariate curve resolution–alternating least squares (MCR-ALS) applied to excitation-emission matrix (EEM) fluorescence data. The results from these different methods were then merged into a single dataset for a global principal component analysis (PCA), which allowed us to identify shared patterns and correlations across methods. The results revealed that low-altitude rivers showed the highest DOM content, followed by mid-altitude rivers, while high-altitude rivers presented the lowest. The PCA model indicated that low-frequency regions in the EIS spectra correlated with higher DOM content, whereas mid- to high-frequency regions were associated with lower DOM levels. These frequency-dependent patterns reflected differences in charge transfer and dielectric behavior of DOM in the river samples, which are not accessible through optical techniques. This highlights the potential of EIS as a complementary tool that provides electrochemical information on DOM composition for better water quality assessment.

**Supplementary Information:**

The online version contains supplementary material available at 10.1007/s10661-025-14489-2.

## Introduction

Water contamination is a pressing global issue with severe implications for human health, ecosystem stability, and economic activities (UN-Water, 2021). Rapid urbanization, industrialization, and agricultural expansion have intensified the presence of organic and inorganic pollutants, accelerating the degradation of surface water quality (UNESCO, 2022). Among the factors influencing water quality, dissolved organic matter (DOM) plays a significant role. DOM is a complex mixture of organic molecules originating from plant decomposition, microbial activity, and anthropogenic sources like industrial and agricultural runoff (Fellman et al., 2010).

DOM serves as a carbon source for microorganisms, regulates trophic interactions, and influences the biogeochemical cycling of carbon, nitrogen, and phosphorus (Jansen et al., 2014). DOM can also interact with pollutants through various mechanisms, including complexation, adsorption, and co-transport. For example, DOM contains functional groups such as carboxyl, hydroxyl, and phenolic moieties that can chelate heavy metals like copper, lead, or mercury (Hg^2^⁺), forming stable DOM-metal complexes (Yamashita & Jaffe 2008). Similarly, DOM can bind to hydrophobic organic contaminants, including endocrine-disrupting compounds like bisphenol A (BPA) and nonylphenol, altering their bioavailability and transport in aquatic environments (Gan et al., 2019; Gao et al. 2022). During water treatment, DOM interacts with disinfectants like chlorine, generating harmful disinfection by-products such as trihalomethanes (THMs) and haloacetic acids (HAAs), which pose risks to human health and the environment (Andersson et al., 2020). DOM composition and concentration fluctuate widely depending on vegetation, climate, and human activities (Gad et al.,^2024^).

Generally, terrestrial DOM comes from plant litter and soil organic matter, which is enriched in high-molecular-weight humic and fulvic substances, aromatic compounds, and phenolic groups. In contrast, autochthonous DOM in aquatic environments comes primarily by algae, aquatic plants, and microbial activity and often contains more low-molecular weight proteins, peptides, carbohydrates, and algal exudates (Murphy et al., 2008; Ren et al., 2024). However, soil algae and aquatic plants can also contribute humic-like materials, while microbial decomposition and photochemical transformations can convert labile proteins and sugars into more recalcitrant and aromatic compounds. These properties are further shaped by biogeochemical processes such as microbial degradation, photodegradation, and adsorption onto particulate matter (Song et al., 2022). Given its widespread distribution and dynamic behavior, understanding and monitoring the sources, composition, and transformations of DOM is essential for effective water resource management and the preservation of water quality and safety (Dean & Battin, 2024).

DOM analysis traditionally relies on chemical indicators such as chemical oxygen demand (COD), biochemical oxygen demand (BOD), dissolved organic carbon (DOC), and total organic carbon (TOC) (Aguilar-Torrejón et al., 2023). However, these methods are limited in their ability to provide detailed insights into the molecular composition and reactivity of DOM. To address these limitations, advanced analytical techniques have been applied for DOM characterization. Chromophoric dissolved organic matter (CDOM), a subset of DOM, can be characterized by both ultraviolet–visible (UV–Vis) molecular absorption and fluorescence spectroscopies, due to its aromatic and conjugated structures with a particular emphasis on excitation-emission matrix (EEM) fluorescence spectroscopy (Kowalczuk et al., 2005). This technique provides a three-dimensional fluorescence landscape that helps to distinguish overlapped fluorophores and to identify specific DOM components based on their spectral signatures for detailed compositional analysis.

Spectroscopic methods, particularly UV–Vis molecular absorption and fluorescence spectroscopy, are widely used detailed insights into the optical properties of DOM. UV–Vis molecular absorption spectroscopy, especially the measurement at 254 nm, serves as a reliable proxy for the aromaticity and molecular size of DOM (Li & Hur, 2017). This approach is effective for assessing bulk DOM properties and tracking its variations during water treatment processes. In contrast, fluorescence spectroscopy provides more detailed information by determining both qualitative and quantitative indices, such as the fluorescence index (FI), biological index (BIX), and humification index (HIX). Fluorescence spectroscopy also identifies specific DOM components, including humic-like and protein-like substances (Fellman et al., 2010; McKnight et al., 2001; Ohno, 2002). The excitation-emission matrices (EEMs) generated through fluorescence analysis contain extensive information about DOM composition, potential sources, and transformation processes (Marcé et al., 2021; Murphy et al., 2011).

UV–Vis molecular absorption and fluorescence spectroscopies provide mainly qualitative and semiquantitative information. Although specific fluorescent DOM components (FDOM) can be tracked quantitatively, these techniques may not fully capture the chemical and electrochemical complexity of DOM, especially the non-chromophoric fraction that lacks optical activity. These optical techniques are widely used for water quality monitoring, including real-time applications with in-line UV–Vis and fluorescence DOM sensors. However, they may still face certain limitations, such as limited selectivity in UV–Vis molecular absorption and reduced sensitivity to non-chromophoric components. Fourier transform ion cyclotron resonance mass spectrometry (FT-ICR MS) and liquid chromatography coupled to high-resolution mass spectrometry (LC-HRMS) have emerged as powerful tools for molecular-level characterization of DOM, allowing the identification of thousands of individual compounds (Ding et al., 2024; Patrone et al., 2024). However, the high cost and complexity of MS, along with the need of advanced technical skills and expertise to conduct the analysis, limit its widespread adoption, particularly in routine monitoring applications. Nuclear magnetic resonance (NMR) is also applied for assessment of molecular-level information of DOM. Although traditionally limited by low sensitivity and overlapping signals, recent advances in instrumentation, data analysis, and sample preparation have improved its resolution and applicability (Mitschke et al., 2023).

Overall, these techniques provide valuable insights into DOM composition with specific limitations in terms of sensitivity, selectivity, cost, or accessibility. In the literature, the electrochemical behavior of DOM remains underexplored, despite its structural complexity and the wide range of molecules it contains and which differ in charge, polarity, and redox activity. Recent advancements in sensor technologies, particularly those based on electrochemical methods, have opened new possibilities for real-time, in situ monitoring of DOM (Lawal, 2023).

Electrochemical Impedance Spectroscopy (EIS) measures the impedance of a system over a range of alternating current frequencies (Lvovich, 2014). In aqueous solutions, EIS detects how an applied electrical signal is modulated by the physicochemical properties of the medium, including ionic conductivity, dielectric constant, and interfacial charge-transfer resistance. These properties vary depending on the concentration and nature of dissolved organic and inorganic compounds (Bonanos et al., 2018). In this context, EIS provides complementary information for DOM analysis by capturing frequency-dependent changes in the electrical behavior of water samples, which may reflect the presence, composition, and molecular characteristics of DOM. Other parameters, like the presence of nutrients in the solution may also have an effect on EIS results, so the method can reveal changes due to the presence of DOM in known conditions of the other parameters. This approach has a potential high sensitivity but a limited specificity.

While UV molecular absorption and fluorescence spectroscopies remain the standard methods for DOM characterization in the laboratory, portable UV and fluorometric instruments have also proven effective for field monitoring (Carstea et al., 2020). EIS offers an additional tool for real-time analysis, with sensitivity to electrochemical features that are not accessible through optical techniques. Moreover, EIS can be adapted for in situ applications, making it suitable for continuous environmental monitoring. This supports our hypothesis that EIS adds value to DOM analysis by addressing aspects of its behavior that are not covered by classical spectroscopic methods.

Given the complex nature of DOM, integrating data from multiple analytical techniques is essential for a more comprehensive understanding. However, the large volume of data generated by UV–Vis molecular absorption, fluorescence spectroscopy, and EIS requires the use of additional tools for better interpretation. Chemometrics and multivariate data analysis (MDA) methods are specifically designed to handle complex datasets by reducing data dimensionality, uncovering patterns, trends, and correlations between variables (insights that would otherwise be difficult to unveil using traditional univariate analysis).

Chemometrics applies multivariate statistical methods to extract meaningful information from large and complex datasets. In the context of DOM analysis, chemometrics enables the individual analysis of physicochemical parameters, fluorescence indices, resolved DOM fractions, and spectral data from techniques such as UV–Vis or EIS. In addition, chemometrics facilitates the simultaneous interpretation of combined data from physicochemical sensors, UV–Vis molecular absorption, fluorescence, and EIS. This data aggregation strategy allows for a more detailed characterization of DOM fractions by applying bi- and trilinear decomposition multivariate methods such as PCA, parallel factor analysis (PARAFAC, Murphy et al., 2013) and multivariate curve resolution by alternating least squares (MCR-ALS, Zhang et al., 2014). These can extract relevant features and quantitative patterns from the water temperature, pH, conductivity, UV–VIS molecular absorption spectra, fluorescence EEM matrix and EIS spectra, shedding light on properties such as composition, sources, and the temporal or spatial behavior of DOM (Jaffé et al., 2008; Zhang et al., 2014). On the one hand, physicochemical sensors, UV–VIS molecular absorption and fluorescence have been widely used in combination with chemometric techniques to study DOM in environmental contexts (Vera et al., 2017; Zhang et al., 2014). On the other hand, the combination of EIS and chemometric data analysis techniques, while not yet widely adopted, has shown potential and has been applied in various fields, including environmental monitoring and energy storage (Buchicchio et al., 2023; Du et al., 2023; Serrano-Pallicer et al., 2018).

Therefore, this study aims to evaluate whether EIS, together with classical techniques such as UV–Vis absorption, fluorescence spectroscopy, and basic water quality parameters (pH, conductivity, temperature), can provide complementary information for the characterization of DOM in river water. The starting hypothesis is that EIS can reveal electrochemical features such as the frequency dependent dielectric behavior of DOM as a function of its composition that are not fully captured by optical methods. To evaluate this, chemometric tools are applied to analyze each dataset individually and also to combine their results. Therefore, the pursued objectives are to describe DOM composition across rivers using different techniques and to examine the relationship between the resolved optical and electrochemical responses.

## Material and methods

### Sampling

**Fig. 1:**
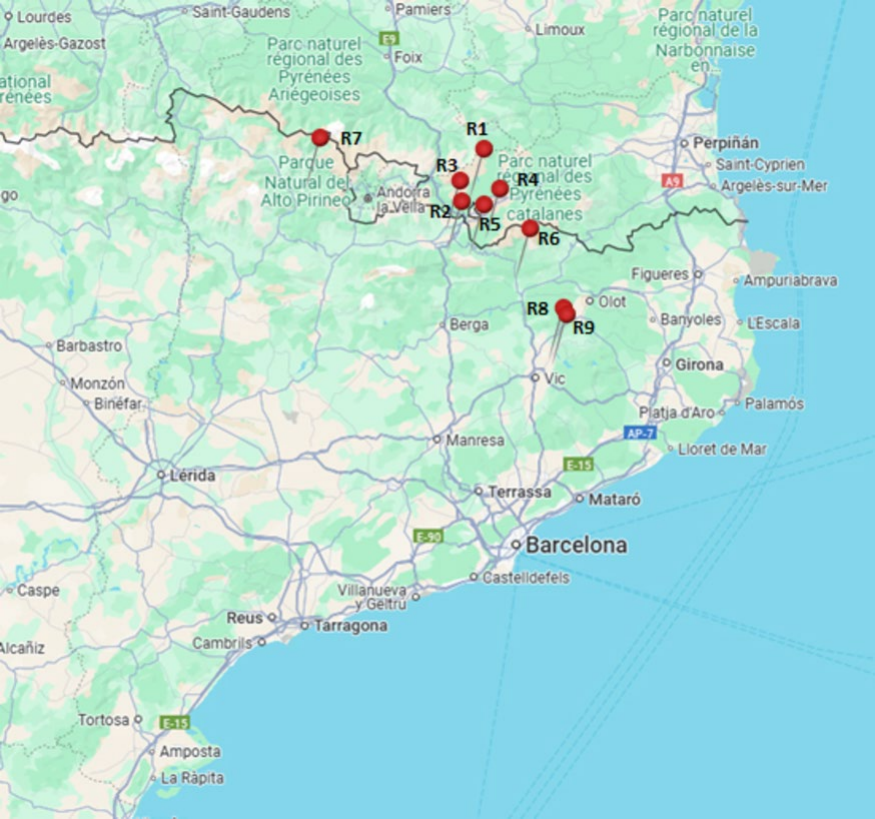
Geographic location of the water sampling points across the Spanish and French Pyrenees. Red pins indicate the sampling sites included in this study. R1 Angostrina river; R2 Font Freda river; R3 Segre river; R4 Riu de la Vila river; R5 Riu D’Alp river; R6 Freser river; R7 Cassibros river; R8 Ter river; R9 Ter river. For more details, see Supplementary Table 1: River sample characteristics and their potential environmental impacts

During the spring 2023 campaign, 9 water samples (see Supplementary Table 1 for detailed description) were collected at 9 locations from eight different rivers in the Catalan-French Pyrenees (see Fig. 1). These sampling locations were selected across different altitudes (at low-, mid-, and high-altitude mountainous regions) to represent varying DOM content and capture a range of environmental and anthropogenic influences, including industrial, agricultural, and tourism activities, as well as habitats with minimal or no human impact. Three physicochemical parameters (water temperature, pH, and conductivity) were measured in situ at the moment of sampling using a portable sensor from Hanna Instruments (Hanna HI98190,Woonsocket, USA).

All water samples were collected and stored in glass bottles at low temperatures (4 °C) during transport to the laboratory. The samples were then stabilized at room temperature (20 °C) before analysis. For DOM analysis, the samples were filtered using polyvinylidene difluoride (PVDF) membrane syringe filters (0.45 μm) to remove particles and sand. Milli-Q water was used both as a blank reference for baseline correction in UV–Vis and fluorescence spectroscopies. Also, Milli-Q water has been used as a control sample to represent DOM-free water in comparative analyses.

### UV–vis molecular absorption and fluorescence spectroscopies

UV–Vis molecular absorption spectra of the water samples were recorded using an HP-Agilent 8453 spectrophotometer (Agilent Technologies, CA, USA), with a diode array detector (DAD). Measurements covered 190–1100 nm (scan rate: 1 nms^−1^, time response: 1 s, spectral bandwidth: 1 nm), using a 1 cm quartz cuvette at room temperature (20 ºC). Absorbance at 254 nm (**uv254**) was used as a proxy for DOM aromaticity (Li & Hur, 2017). Data acquisition and processing were performed using the UV Spectrophotometer ChemStation B.02.01 software.

Fluorescence excitation-emission matrices (EEMs) were recorded using a Cary Eclipse Fluorescence Spectrophotometer (Agilent Technologies, CA, USA). Excitation wavelengths ranged from 230 to 450 nm (5 nm increments), and emission wavelengths ranged from 250 to 550 nm (1 nm increments). Measurements were performed at a scan rate of 1200 nm min^−1^ with a PMT voltage of 600 V. Rayleigh and Raman scattering were corrected before chemometric analysis to mitigate undesirable non-linear effects following Zepp et al. (2004). A screening of each sample for potential inner filter effect based on UV absorbance at 254 nm was done. Samples with absorbance exceeding 0.05–0.10 cm⁻^1^ were diluted with Milli-Q water until absorbance dropped below this threshold, minimizing the risk of an inner filter effect as recommended by Ohno (2002). Inner filter effect corrections were unnecessary for mountain water samples (showing neither high optical density nor turbidity) but were applied to R3, R8 and R9 samples (UV absorbance at 254) by diluting with Milli-Q water. Fluorescence intensity was normalized to quinine sulfate units (QSU), where 1 QSU represents the maximum fluorescence intensity of 0.01 mg L^−1^ quinine in 1 N H₂SO₄ at excitation and emission of 350 nm and 450 nm, respectively (Zhang et al., 2009a, 2009b). Data acquisition was performed using Cary WinFLR v1.2 software, and further processing carried out in MATLAB® 2024a (MathWorks Inc., Natick, MA, USA) for chemometric analysis.

### Electrochemical impedance spectroscopy (EIS) device

The impedance spectra of developed capacitive devices were recorded to evaluate the influence of DOM content in water on their impedance parameters. The devices consisted of interdigitated aluminum (Al) comb electrodes and an air-voided aluminum oxide (Al_2_O_3_) layer. The fabrication process starts with the production of the interdigitated electrodes by means of thermal evaporation and photolithography processes. Subsequently, polystyrene (PS) nano and microparticles, which acts as sacrificial layer, are deposited by electrospray, and an Al_2_O_3_ layer is grown by atomic layer deposition (ALD). Finally, the PS particles are carbonized.

**Fig. 2:**
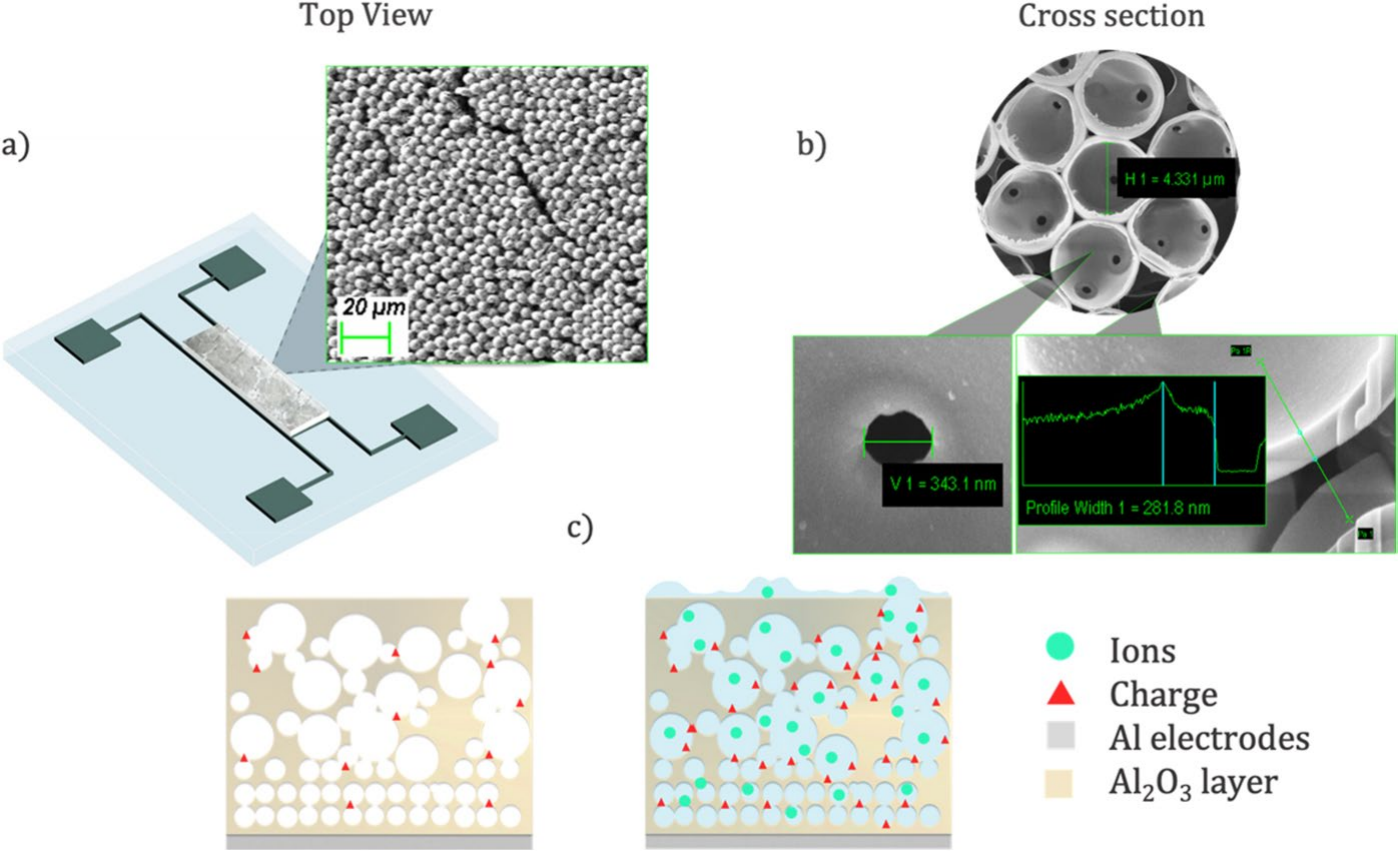
Scanning electron microscope (SEM) and focused ion beam (FIB) images of the Al₂O₃ air-voided capacitive devices and their proposed operation principle. (**a**) Design and top surface view at 20 µm resolution; (**b**) cross-section of the air-voided Al₂O₃ layer at different resolutions; (**c**) schematic representation of the device operation, showing the Al₂O₃ air-voided capacitive device without water (left) and with water (right)

Figure 2 displays a graphic representation of the final devices as well as the resulting air- Al_2_O_3_ layer. The ceramic layer reveals a close packed face-centered cubic (FCC) structure with a typical thickness of several hundreds of micrometers (Fig. 2a). In particular, scanning electron microscope (SEM) images (Fig. 2b) reveal that the air-voided layer contained spherical pores of approximately 5 µm and 0.5 µm in diameter, separated by Al_2_O_3_ walls measuring 250–300 nm in thickness.

Figure 2c illustrates the proposed hypothesis on how DOM content affects the impedance spectra of the fabricated devices. The electrical impedance characteristics of the Al_2_O_3_ air-voided capacitive devices were measured to assess their response to different water samples.

EIS measurements were conducted at room temperature using a HIOKI IM3590 (HIOKI E.E. CORPORATION, Japan) impedance analyzer over a 0.1 Hz—200 kHz frequency range with a 10 mV sinusoidal signal with amplitude and no bias voltage. Measurements were conducted under wet conditions, with 20 µL water samples deposited via drop-casting onto the capacitive device’s active area. In particular, five water samples were analyzed: DOM-free and four river water samples (R3, R5, R6 and R9).

To ensure reproducibility, two replicates per sample were obtained by recording the EIS spectra twice under identical conditions. First, impedance spectra were measured immediately after sample deposition of the aqueous sample on the device surface. Then, after the device dried completely, the same water sample was reapplied, and a second EIS spectra was recorded. This approach provided replicates for comparative analysis.

### Data analysis

**Fig. 3:**
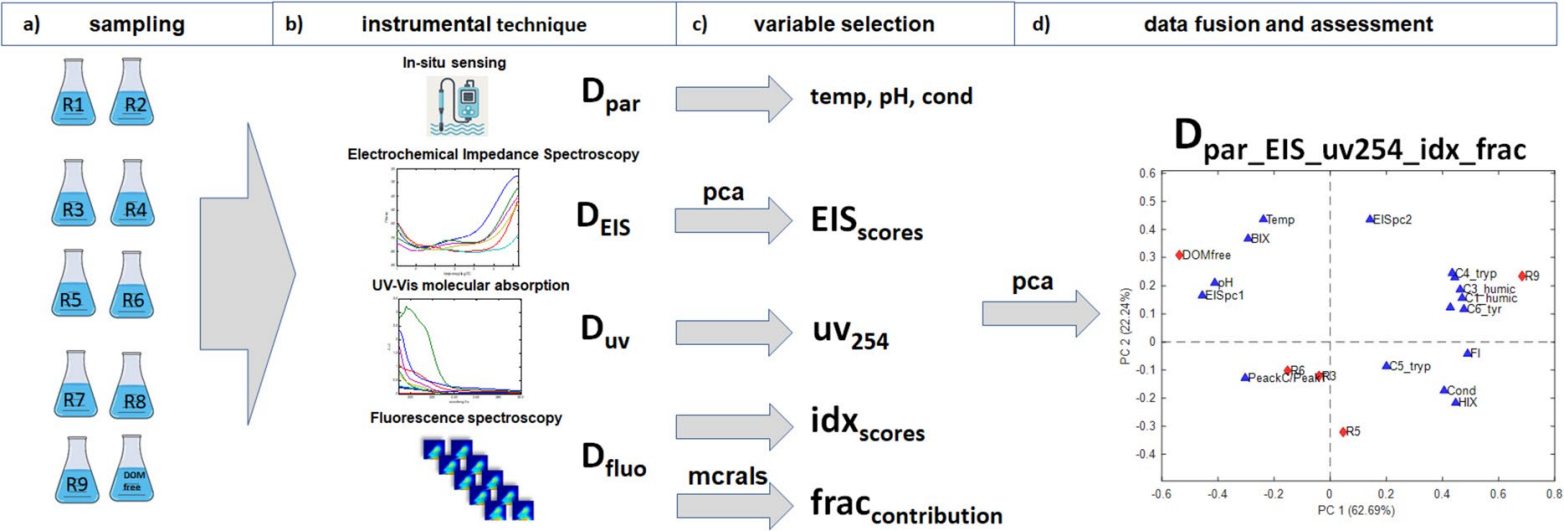
Methodological pipeline of EIS integration and results confirmation. R1-R9 river water samples used in this study. Milli-Q water also used as DOM-free water sample. **D**_**par**_ – matrix containing temperature, pH and conductivity vectors of the ten water samples; **D**_**EIS**_—matrix containing EIS spectra of the ten water samples; **D**_**UV**_ matrix containing UV spectra;; **D**_**fluo**_ matrix containing ten water fluorescence excitation- emission matrices. **EIS**_**scores**_ – two principal component scores variables derived from PCA of the EIS matrix; **uv**_**254**_—a vector of absorbance values of the ten samples taken at 254 nm; **idx**_**scores**_ – a matrix containing fluorescence indexes calculated for the ten water samples; **frac**_**contribution**_ – a matrix containing the six relative concentration contributions for MCRALS resolved components; **D**_**par_eis_uv254_idx_frac**_—a matrix of all aggregated variables in the variable selection process

The methodological pipeline shown in Fig. 3 illustrates the sequential workflow applied in this study. In Panel A, ten water samples were collected and analyzed for physicochemical parameters (temperature, pH, and electrical conductivity) in situ using portable instruments. In Panel B, samples were subsequently analyzed using the three proposed instrumental techniques: EIS, UV–Vis molecular absorption and fluorescence spectroscopies. The resulting datasets were organized into four matrices: **D**_**par**_ (physicochemical parameters), **D**_**EIS**_ (EIS spectra), **D**_**UV**_ (UV–Vis spectra), and **D**_**fluo**_ (fluorescence data). In Panel C displays the variable selection process.** D**_**EIS**_ dataset was analyzed using PCA to extract systematic variation through principal component scores (EIS score variables), enabling the preliminary differentiation of river samples based on their DOM composition. Simultaneously, the **D**_**UV**_ dataset provided bulk optical properties such as absorbance at 254 nm (**uv**_**254**_ variable), used as a proxy for DOM load. In parallel, the **D**_**fluo**_ data was used to calculate fluorescence indices (FI, HIX, BIX, Peak C/Peak T – **idx** score variables). Further details about these indices are provided in the Supplementary Material. The same dataset also enabled decomposition of EEMs into distinct DOM fractions and to calculate their corresponding relative concentrations (**frac** relative contributions) using MCR-ALS. Finally, in Panel D, results from all techniques were integrated into a single data matrix, **D**_**par_eis_uv254_idx_frac**_**,** combining physicochemical parameters, EIS-derived scores, uv254, fluorescence indices, and MCR-ALS fractions. This fused dataset was subjected to a new PCA to synthesize information and assess DOM variability across samples.

#### PCA

PCA is a widely used bilinear decomposition method in multivariate statistical analysis, particularly in environmental data studies. PCA assumes that most of the variance of the original dataset is explained by a small number of orthogonal factors (principal components), (Jolliffe, 2011). In this study, PCA was applied in two contexts: 1) PCA of the EIS dataset to differentiate river water samples based on their impedance spectra and DOM composition, and 2) PCA of the ‘fused’ dataset to integrate and correlate complementary information from physicochemical sensors, EIS, UV–Vis molecular absorption and fluorescence spectroscopy.

### PCA of EIS single dataset

To investigate water quality differences, PCA was first applied to the EIS spectral dataset (**D**_**eis**_), structured as a 10 × 500 matrix where rows (*nr* = 10) represented five water samples (each with two replicates: Milli-Q, R3, R5, R6 and R9) and columns (*nv* = 500) corresponded to the 500 EIS spectral phase angles measured in the 0.1—20,000 Hz frequency range after their logarithmic (log10) transformation. Before PCA, EIS data was smoothed using a Savitzky–Golay filter (0th order, 15 points per window) and mean-centered. This preliminary investigation is performed to test the performance of EIS to distinguish among different river water samples. The **D**_**eis**_ dataset was decomposed using the PCA bilinear model according to the following Equation:

1 $${\mathbf{D}}_{\mathbf{e}\mathbf{i}\mathbf{s}}= {\mathbf{T}}_{\mathbf{e}\mathbf{i}\mathbf{s}}{{\mathbf{P}}^{\mathbf{T}}}_{\mathbf{e}\mathbf{i}\mathbf{s}}+{\mathbf{E}}_{\mathbf{e}\mathbf{i}\mathbf{s}}$$

where **T**_**eis**_ is the scores matrix with dimensions *nr* x *nc*, showing the projection of the *nr* samples onto the *nc* principal components and the **P**^**T**^_**eis**_ is the loadings matrix, *nc* x *nv*), giving he contribution of the *nv* different frequency regions of the EIS spectrum to each of the *nc* principal components, highlighting what EIS spectral regions are associated with high and low DOM content. Finally, the matrix **E**_**eis**_ (10 × 500) (residual matrix, *nr* x *nv*) has the unexplained data variance and the same dimensions as **D**_**eis**_**.**

### PCA of the fused dataset

PCA is also applied to characterize DOM using different analytical techniques and to correlate previously obtained EIS results, with temperature, pH, conductivity water quality parameters, UV254 absorbance fluorescence indices and DOM UV, and fluorescence characterization parameters. This aggregated dataset, **D**_**par_eis_uv254_idx_fra,c**_ was arranged by horizontal concatenation including a) the three physicochemical parameters (size 5 × 3); b) the two principal component scores from the D_EIS_ PCA model (**T**_**eis**_ matrix, size 5 × 2) averaged across replicates; c) the **uv**_**254**_ absorption values (size 5 × 1), d) the fluorescence indices (FI; HIX, BIX, PeakC/PeakT from the **idx** dataset, size 5 × 4), e) the MCR-ALS resolved DOM fractions (see below the results of the Multivariate Curve Resolution analysis **of** the **frac** dataset (size 5 × 6). As a result, the horizontally concatenated fused data matrix (**D**_**par_eis_uv254_idx_frac**_) had 5 rows and 16 variables. This dataset was autoscaled before performing PCA.

A new PCA model was then generated according to decomposition Eq. 2:

2 $${\mathbf{D}}_{\mathbf{p}\mathbf{a}\mathbf{r}\_\mathbf{e}\mathbf{i}\mathbf{s}\_\mathbf{u}\mathbf{v}254\_\mathbf{i}\mathbf{d}\mathbf{x}\_\mathbf{f}\mathbf{r}\mathbf{a}\mathbf{c}}={\mathbf{T}}_{\mathbf{p}\mathbf{a}\mathbf{r}\_\mathbf{e}\mathbf{i}\mathbf{s}\_\mathbf{u}\mathbf{v}254\_\mathbf{i}\mathbf{d}\mathbf{x}\_\mathbf{f}\mathbf{r}\mathbf{a}\mathbf{c}} {{\mathbf{P}}^{\mathbf{T}}}_{\mathbf{p}\mathbf{a}\mathbf{r}\_\mathbf{e}\mathbf{i}\mathbf{s}\_\mathbf{u}\mathbf{v}254\_\mathbf{i}\mathbf{d}\mathbf{x}\_\mathbf{f}\mathbf{r}\mathbf{a}\mathbf{c}}+{\mathbf{E}}_{\mathbf{p}\mathbf{a}\mathbf{r}\_\mathbf{e}\mathbf{i}\mathbf{s}\_\mathbf{u}\mathbf{v}254\_\mathbf{i}\mathbf{d}\mathbf{x}\_\mathbf{f}\mathbf{r}\mathbf{a}\mathbf{c}}$$

Here, **T**_**par_eis_uv254_idx_frac**_ matrix are the new sample scores matrix showing their variability on the new PC axes and **P**^**T**^_**par_eis_uv254_idx_fra**_ describe the correlations between EIS-derived features, water physicochemical properties and DOM properties, linking specific impedance spectral regions with DOM fractions and fluorescence indices. Finally, **E**_**par_eis_uv254_idx_frac**_ represented the unexplained residual variance in the system.

PCA calculations were performed using PLS Toolbox 9.2 (Eigenvector Research, Manson, WA, USA) in MATLAB 2024b (MathWorks Inc., Natick, MA, USA).

### Multivariate curve resolution analysis

Multivariate Curve Resolution (MCR) is a chemometric method used to decompose complex datasets into pure component profiles. It is based on a bilinear factor decomposition model, analogous to the multivariate (multi-sample and multi-wavelength) generalization of Lambert–Beer’s law. When applied to spectral data, MCR enables the resolution of overlapping spectral features, facilitating the characterization of complex chemical mixtures, like those present in DOM. Moreover, by resolving spectral contributions, MCR enhances the interpretation of chemical data, providing valuable insights into DOM sources, transformations, and compositional variations.

Here, MCR was applied to resolve EEM fluorescence data. Each individual water sample produced a single data matrix, **D**_**fluo**_**,** with 45 rows (excitation wavelengths) and 301 columns (emission wavelengths). The bilinear factor decomposition model for the single data matrix, **D**_**fluo**_**,** is expressed as:

3 $${\mathbf{D}}_{\mathbf{f}\mathbf{l}\mathbf{u}\mathbf{o}}\text{ = }{\text{S}}_{\text{ex}}{{\text{S}}}_{\text{em}}^{\text{T}}+ \text{E}$$

where **S**_**ex**_ (45 × nc) is the excitation factor matrix, for the *nc* resolved components, and **S**^**T**^_**em**_ (*nc* × 301) is the emission factor matrix, of the same resolved components, and **E** (same size as **D**_**fluo**_) is the residual matrix describing the variance not explained by the bilinear model.

The MCR bilinear model described by Eq. 3 is usually solved using a sequential process comprising three key steps. First, the number of components (*nc*) is estimated using the singular value decomposition (SVD) to identify the significant chemical or spectral contributions. Next, an initial estimate of either **S**_**ex**_ or **S**_**em**_ is obtained by identifying the most distinct (columns or rows) features through a pure variable detection approach (Windig & Stephenson, 1992). Finally, Eq. 3 is solved using an alternating least squares (ALS) iterative optimization (see Tauler 1995; de Juan & Tauler, 2001), under non-negativity constraints for the two factor matrices, **S**_**ex**_ or **S**_**em**_, to ensure physically meaningful solutions. The MCR-ALS iterative process converges rapidly, typically within a few iterations, providing optimized profiles (in this case, related to fluorescence excitation and emission spectra).

When only non-negativity constraints are applied during the ALS optimization, the bilinear decomposition of a single EEM data matrix (single sample), **D**_**fluo**_**,** into the excitation, **S**_**ex**_**,** and emission, **S**_**em**_ does not guarantee a unique solution. In contrast, when multiple EEMs datasets (matrices) from the same system at different conditions are analyzed simultaneously, the bilinear model described in Eq. 3 can be extended to a trilinear model, as shown in Eq. 4 (Tauler 1995; de Juan & Tauler, 2001, Tauler 2020). In this case, *k* represents the considered river water sample analyzed (in this case, ranging from R1 to R9 and the blank sample).

4 $${{\mathbf{D}}_{\mathbf{k}}\text{ = }{\text{S}}_{\text{ex}}{\mathbf{C}}_{\mathbf{k}}{\text{S}}_{\text{em}}^{\text{T}}+ \text{ E} }_{\mathbf{k}}\boldsymbol{ }k=1,\dots ,10$$

In the trilinear model described by Eq. 4, the resolved excitation fluorescence spectra (**S**_**ex**_, 45 × *nc*) and emission fluorescence spectra (**S**_**em**_, *nc* × 301) remain constant across all analyzed samples (*k* = 1, …,10). However, the **C**_**k**_ matrices which differ for each **D**_**k**_ dataset contain the relative quantitative information for each resolved component (*nc*) in each sample (*k*). These **C**_**k**_ matrices (*nc* x *nc*) are represented as diagonal matrices, where the values indicate the relative contributions (or concentrations) of each component (i.e., the number of resolved DOM fractions across the 10 water samples, as described in Eq. 4). The simultaneous trilinear factor decomposition of all **D**_**k**_ matrices yields unique solutions under mild (non-negativity) constraints, enhancing the robustness of the analysis. The trilinear model is implemented algorithmically during the ALS optimization as a constraint. For detailed information on the implementation and applications of the trilinearity constraint, refer to Tauler et al., (1995), de Juan and Tauler (2001) and Tauler (2020). The application of the MCR-ALS trilinear factor decomposition to EEM data produces analogous results to the application of the PARAFAC method as shown in previous works (Tauler et al., 1998; Zhang et al., 2014).

The quality of the MCR-ALS optimization was assessed using the explained data variance (R^2^), calculated as:

5 $${\text{R}}^{2}=100\times (1-\frac{\sum_{\text{k}=1}^{10}\sum_{\text{i}=1}^{45}\sum_{\text{j}=1}^{301}{\left( {\text{d}}_{\text{ijk}}-{\widehat{\text{d}}}_{\text{ijk}} \right)}^{2}}{\sum_{\text{k}=1}^{10}\sum_{\text{i}=1}^{45}\sum_{\text{j}=1}^{301}{\text{d}}_{\text{ijk}}^{2}} )$$

where, **d**_ijk_ represents experimental values in the data matrix, and ${\widehat{\text{d}}}_{\text{ijk}}$ are the corresponding MCR-ALS estimated values using the trilinear model (Eq. 4).

While split-half validation was not feasible due to the small (n = 10) sample size (Murphy et al., 2013), the robustness of the MCR-ALS model was ensured by examining the residuals and the spectral features of the resolved components. The interpretability of the excitation and emission spectra, together with their coherence with profiles reported in the literature, supports the reliability of the model outcomes.

The MCR-ALS Toolbox (Jaumot et al., 2015; available at http://www.mcrals.info/) and MATLAB 2024b release (The Mathworks Inc., MA, USA) were used for the calculations.

## Results and discussion

The aim of this study was to evaluate whether integrating EIS with classical spectroscopic and physicochemical techniques can bring new information and improve the characterization of DOM in river water samples. The analysis started with EIS spectra to assess whether electrochemical differences could help differentiate the samples based on DOM-related properties. Physicochemical parameters, UV–Vis molecular absorption, and fluorescence spectroscopy were then used to complement these results and provide additional information on DOM composition. Finally, all variables were combined in a global multivariate framework using PCA to explore DOM variability across rivers influenced by different environmental and anthropogenic conditions.

### Rivers samples characterization using EIS spectra

The potential use of EIS spectra as a tool for distinguishing water samples of varying quality was evaluated using PCA. By reducing the spectral data into principal component scores (EIS scores variables), this analysis provided preliminary insights into differences among the river samples, primarily driven by variations in their electrochemical characteristics.

**Fig. 4:**
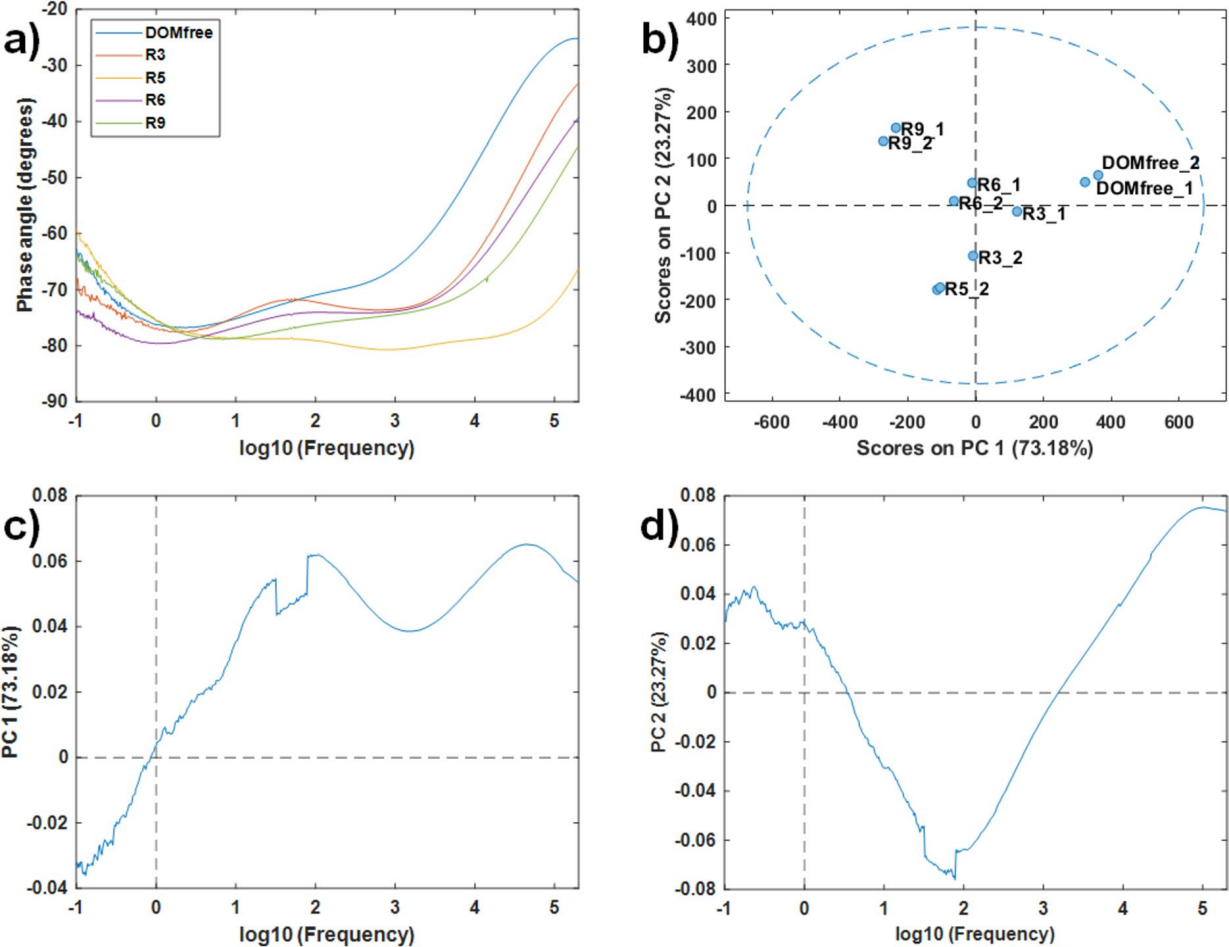
Characterization of river water samples using EIS and PCA: **a**) Bode phase plot of raw EIS spectra; **b**) PCA scores plot (PC1 vs PC2) showing water samples in replicates; **c**) PCA loadings (PC1); **d**) PCA loadings (PC2)

Figure 4a shows the raw EIS spectra recorded for five river water samples and DOM-free sample, measured immediately after sample application.

Among all the analyzed samples, the DOM-free water showed the highest phase angles in the high-frequency region of the Bode phase plot (see Fig. 4a). This response indicated a stronger capacitive behavior (or reduced resistive dominance) at these frequencies for pure DOM-free water compared to the other river water samples.

PCA of the EIS dataset yielded a model with two principal components, explaining a 96% of the total variance (PC1: 73%, PC2: 23%). The PCA scores plot (Fig. 4b) revealed distinct clustering of water samples based on their EIS characteristics. PC1 showed that the R9 (both replicates, variables R9_1 and R9_2) had negative scores, clearly separating these samples from the DOM-free samples (variables DOMfree_1 and DOMfree_2), which had positive scores. This suggested that R9 sample presented a different EIS profile compared to DOM-free. The remaining river samples (R3, R5 and R6) took intermediate positions on PC1, indicating their EIS profiles fall between the extremes represented by R9 and DOM-free samples. PC1 loadings (Fig. 4c) emphasized that the middle to high-frequency ranges contributed most to the variance, likely reflecting differences in sample conductivity and dielectric behavior. DOM rich samples such as R9 generally showed higher ionic content, which increases conductivity, while DOM free sample showed lower conductivity and different capacitive responses in these frequency ranges. PC2 further distinguished R9 samples (positive scores) from R5 samples (negative scores), suggesting additional variability in DOM composition beyond overall charge differences. The PC2 loadings (Fig. 4d) indicated that this component captured variations at the lowest and highest frequency regions characterizing differences within DOM-loaded samples. These variations suggested specific differences in DOM composition, potentially due to variations in their ionic characteristics.

These preliminary results demonstrated that EIS combined with PCA is effective in differentiating water samples based on ionic and electrochemical properties. However, the specific aspects of DOM affecting EIS spectra remained limited. To achieve a more comprehensive characterization of DOM, additional UV–Vis molecular absorption and fluorescence spectroscopies were required as source of complementary chemical information.

### River samples DOM characterization using classical methods

Physicochemical parameters (water temperature, pH and conductivity), UV–Vis molecular absorption and fluorescence spectroscopies are valuable tools for DOM characterization due to their non-destructive nature and low cost. The water temperature, pH, and conductivity usually provide valuable information about the environmental conditions and the ionic composition of water, which can influence DOM characteristics and reactivity (Kellerman et al., 2014). UV absorbance at 254 nm serves as a proxy (Weishaar et al., 2003) for DOM aromaticity and molecular size, offering a comparative assessment of bulk DOM properties across river samples. In addition, fluorescence spectroscopy provides more specific information on DOM composition (Murphy et al., 2008), allowing for the calculation of fluorescence indices and identification of distinct DOM fractions, such as humic-like and protein-like components. These techniques complement EIS by offering chemical insights into the composition of DOM in the analyzed water samples.

### Physicochemical parameters

In-situ measurement of temperature, pH, and conductivity is a common approach for water characterization, as these physicochemical parameters offer insights into environmental conditions, ionic composition, and potential chemical interactions within the water.

Supplementary Table 1 summarizes the physicochemical parameters (temperature, pH, and conductivity) measured for the ten water samples included in this study.

Water temperature (Temp) ranged from 8.2 °C to 14.5 °C across the river samples, reflecting the winter season environmental conditions at the sampling sites. The highest temperatures were recorded in the low-altitude rivers R8 and R9 (14.2 °C and 14.5 °C, respectively), while the high-altitude rivers (e.g., R4 and R5) showed lower temperatures, consistent with cooler mountainous conditions.

The pH values were ranging from 6.7 to 7.8, indicating slightly acidic to neutral conditions. The lowest pH was observed in R1 (6.7), which may reflect higher organic input or natural acidification from the catchment area (Ludwig et al., 2007; Strobel et al., 2004) The highest pH was recorded in R7 (7.8), suggesting possible carbonate content buffering or reduced organic acid input.

Conductivity (Cond) values showed significant variation, reflecting differences in ionic composition among the samples. The lowest conductivity was measured in R1 (29.7 µS), consistent with its pristine, high-altitude origin and minimal anthropogenic influence (Marchetto et al., 1995). The highest conductivity was recorded in R8 (1625 µS), followed by R9 (594 µS), highlighting the possible impact of urban runoff and wastewater treatment discharge on these low-altitude rivers (Gold et al., 2019). Intermediate conductivity values were observed in the mid-altitude rivers (R3, R6), indicating moderate levels of dissolved ions, likely influenced by agricultural or geological factors. The DOM-free sample, used as a reference was with a room temperature, neutral pH (7.7), and minimal conductivity (1.7 µS).

### UV–vis molecular absorption spectroscopy

**Fig. 5:**
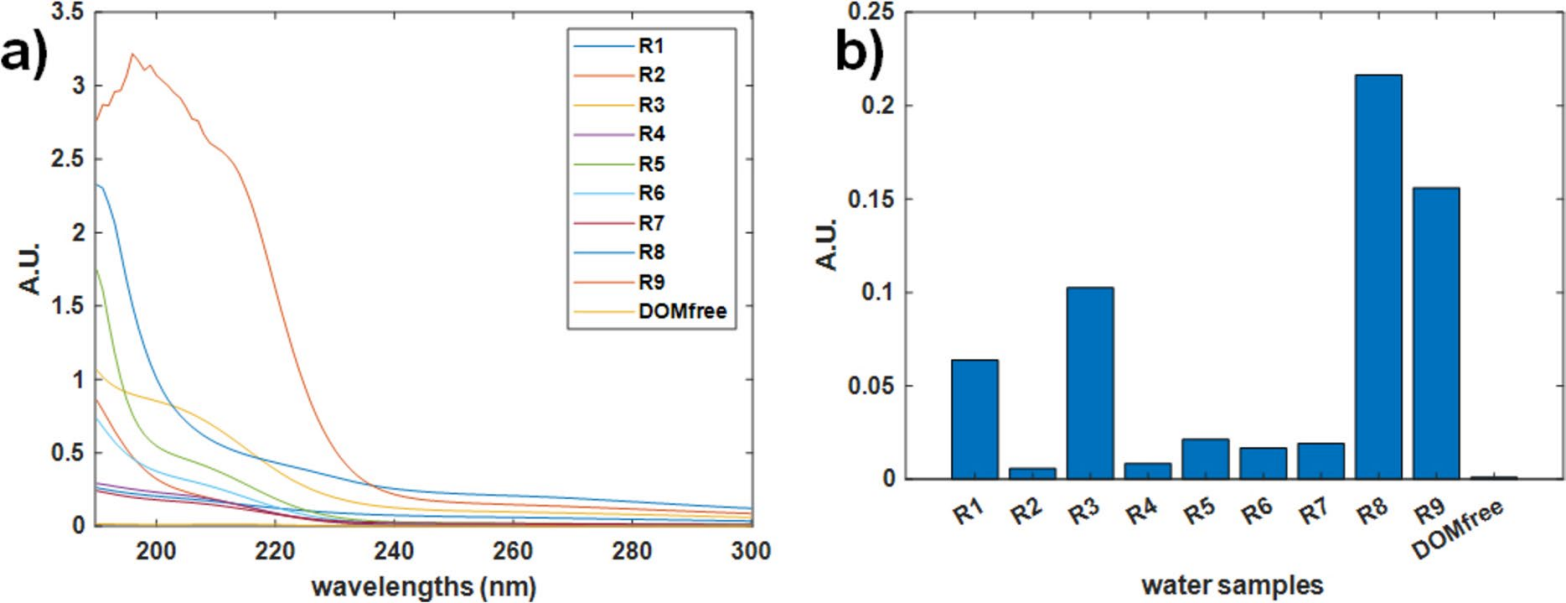
Raw UV–Vis molecular absorption spectra of nine river water samples and the DOM-free reference sample used in this study (a); sample absorbance at 254 nm (b)

Figure 5a shows the raw UV–vis molecular absorption spectra of the ten water samples, while Fig. 5b displays their absorbance at 254 nm. The spectroscopic analysis revealed major trends consistent with the EIS results, particularly those observed in the PCA model. Sample R9 exhibited elevated absorbance in the 200–230 nm range in Fig. 5a, likely due to the presence of nitrate, nitrite, and other UV-absorbing substances associated with urban and agricultural runoff (Sung, 2011). This sample was collected upstream of the wastewater treatment plant, where such contamination could be expected. The R9 sample, which displayed the most distinct profile in the EIS PCA model, presented the second-highest UV254 absorbance, with R8 showing the highest value. This behavior confirmed their higher CDOM load. Both R8 and R9, with high UV254 absorbance, supported the hypothesis that low-altitude rivers were heavily influenced by anthropogenic activities (Coble et al., 2022). Mid- and high-altitude river water samples showed moderate to low UV254 absorbance levels, reflecting their intermediate positions in the DOM gradient based on to the EIS PCA model (e.g., R3, R5, and R6 scores in Fig. 5b).

These UV–Vis molecular absorption spectroscopy results reinforced the EIS findings by confirming the relative DOM distribution across the samples. These results corroborated the significant difference between R9 and DOM-free water samples, as well as the clear distinction between low-altitude river water samples, and mid- and high-altitude river water samples based on their DOM load.

### Fluorescence indices

DOM was further characterized by means of fluorescence measurements. The fluorescence index (FI), humification index (HIX), and biological index (BIX) were calculated for each river water sample to gain insights into DOM sources, degree of humification, and biological activity of the DOM present in these water bodies (see Table 1). In addition, the Peak C/Peak T ratio was used to differentiate DOM components, indicating microbial influence and humic-like characteristics. For further discussion on fluorescence indices, please refer to the Supplementary Material.

**Table 1:** Fluorescence indices calculated to assess DOM composition

River Water Sample	FI	HIX	BIX	PeakC/PeakT
R1	1.86 ± 0.01	0.92 ± 0.04	0.48 ± 0.03	3.1 ± 0.2
R2	1.30 ± 0.01	0.81 ± 0.04	0.60 ± 0.02	0.94 ± 0.07
R3	1.76 ± 0.01	0.71 ± 0.02	0.69 ± 0.07	0.68 ± 0.07
R4	1.43 ± 0.01	0.69 ± 0.01	0.45 ± 0.02	0.84 ± 0.04
R5	1.95 ± 0.00	0.83 ± 0.04	0.71 ± 0.02	1.6 ± 0.1
R6	1.90 ± 0.02	0.70 ± 0.02	0.73 ± 0.04	0.78 ± 0.09
R7	1.99 ± 0.01	0.83 ± 0.03	0.59 ± 0.06	1.5 ± 0.2
R8	2.72 ± 0.04	0.76 ± 0.02	0.88 ± 0.07	1.5 ± 0.1
R9	2.78 ± 0.00	0.87 ± 0.04	0.76 ± 0.03	2.8 ± 0.2
DOM-free	1.24 ± 0.03	0.49 ± 0.07	1.13 ± 0.06	1.00 ± 0.06

FI values were highest in R8 and R9 samples (2.72 and 2.78, respectively), suggesting significant microbiological activity (McKnight et al., 2001) compared to DOM-free and high-altitude river water samples. The elevated Peak C/Peak T ratios (1.54 and 2.75) for these samples further indicated a dominance of humic-like DOM (Coble, 1996), consistent with their distinct separation from the other samples in the EIS PCA model. These results confirmed anthropogenic influences on DOM composition in these low-altitude rivers, likely due to wastewater discharge and agricultural runoff. Additionally, the highest BIX values for R8 and R9 reflected increased autochthonous (Huguet et al., 2009) biological activity compared to other samples.

In contrast, the generally low BIX values (< 0.8) and low HIX values (< 1.0) observed in the mid- and high-altitude rivers indicated limited microbial contribution to DOM and a low degree of humification. These trends aligned with their intermediate positions between R9 and DOM-free in the EIS PCA model, suggesting the presence of terrestrially derived DOM with limited alteration, which could reflect either more persistent material or DOM that has undergone minimal degradation under current environmental conditions.

### DOM fractions identification by MCR-ALS

The application of MCR-ALS for fluorescence data decomposition can offer a significant advantage over traditional fluorescence indices by enabling a more detailed analysis of DOM components (Marín-García & Tauler, 2020; Zhang et al., 2014). While fluorescence indices offer general indicators of DOM origin, MCR-ALS allows for the separation and quantification of distinct fluorophore compounds, giving deeper insights into specific fractions such as humic-like and protein-like substances. This approach is particularly valuable for identifying and quantifying overlapping components within the EEM data, making it more effective for tracking DOM sources.

In this study, MCR-ALS analysis was used to compare DOM fractions across river water samples collected from different altitudes, using Milli-Q water as reference. These results were used to support results from the EIS PCA model.

**Fig. 6:**
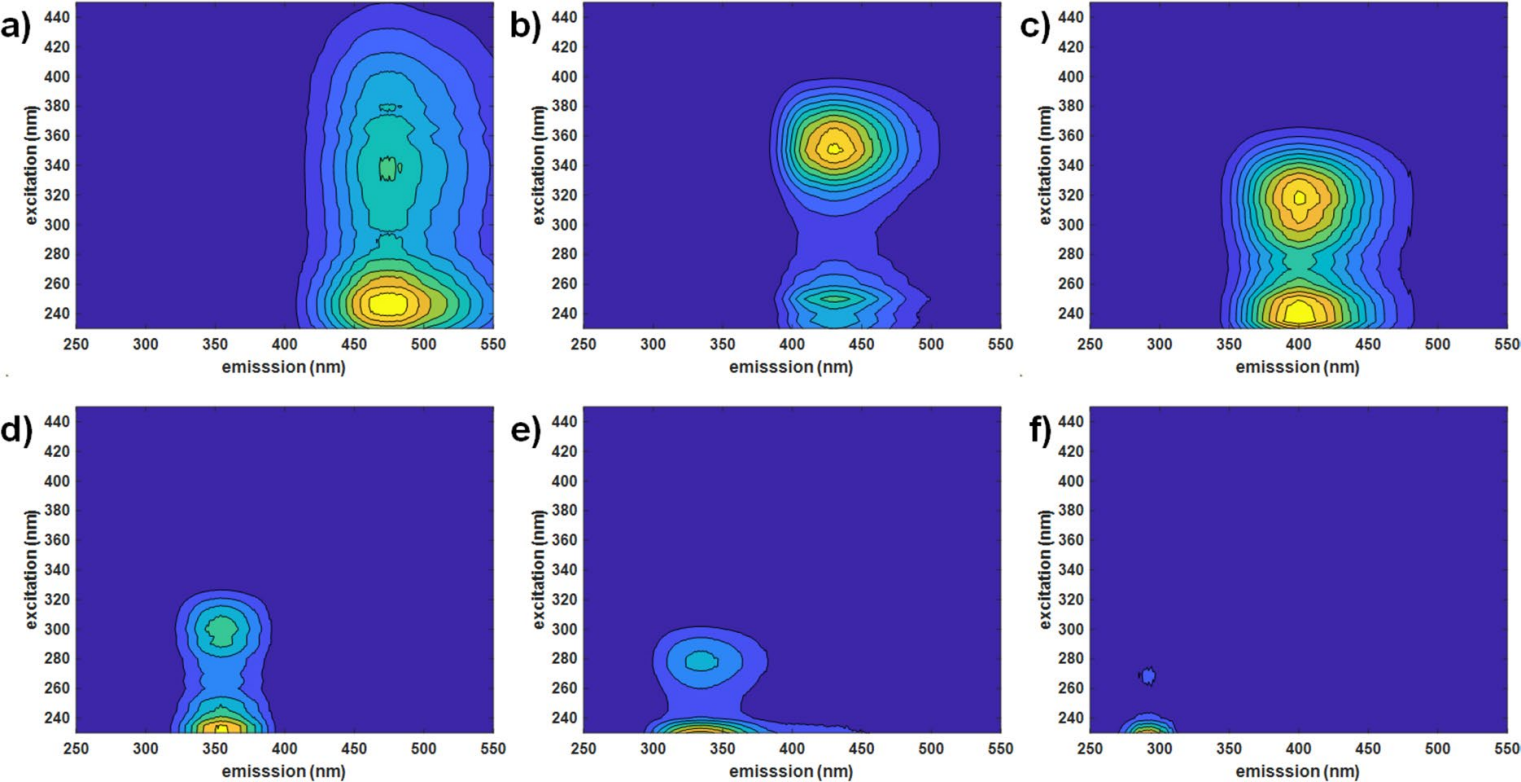
Six MCR-ALS-resolved DOM components (a: C1, b: C2, c: C3, d: C4, e: C5, f: C6) obtained from the analysis of the augmented fluorescence EEM dataset. Reconstructed fluorescence EEMs for each component are shown. Average EEMs were calculated from duplicate measurements of a single representative sample per water type

The MCR-ALS model explained 99.6% of data variance using six components (C1-C6), each representing specific fluorescence characteristics and sources (Fig. 6 a-f and Table 2).

**Table 2:** Summary of DOM fractions (components) identified through MCR-ALS analysis, including fluorescence properties (excitation and emission wavelengths), along with the description of their main biological characteristics

Component (% variance)^a^	λ _Ex/Em wavelength_	Description^b^	Reference
C1 humic-like (38.6)	340 nm (< 260 nm)/ 474 nm	Terrestrial humic-like High-molecular weight humic Highly hydrophobic	Coble, 1996; Stedmon et al., 2003;
C2 humic-like (32.4)	350 nm (< 260 nm)/ 430 nm	Terrestrial/antropogenic humic/fulvic substances More soluble	Coble, 1996; Stedmon & Markager, 2005
C3 humic-like (47.5)	320 nm (< 250 nm)/ 399 nm	Microbial humic-like fluorescence associated with biological activity Low molecular weight	Coble, 1996; Zhang et al., 2009a, 2009b
C4 protein-like (9)	300 nm (< 240 nm)/ 354 nm	Protein Tryptophan-like Less degraded peptide material	Coble, 1996; Mayer et al., 1999
C5 protein-like (16.1)	280 nm (< 240 nm)/ 338 nm	Protein Tryptophan-like Less degraded peptide material	Coble, 1996; Mayer et al., 1999
C6 protein-like (7.9)	270 nm (< 240 nm)/ 294 nm	Protein Tyrosine-like More degraded peptid material	Coble, 1996; Mayer et al., 1999; Yamashita & Tanoue, 2003

^a^Components are categorized by type (humic-like and protein-like), including estimated percentage of variance explained and associated characteristics. The total variance explained by the six MCR-ALS components exceeded 150% due to overlap between their resolved profiles. Unlike in PCA, MCR-ALS components are not orthogonal, meaning some variance can be shared among them. This behavior reflects the natural overlap in sources of variance within the data, as components capture common features across the spectral profiles. This non-orthogonality allows MCR-ALS to resolve components even when they share similar spectral characteristics

^b^Component descriptions reflect molecular weight, hydrophobicity, and degradation level, providing insights into likely sources and the degree of DOM transformation, according to the literature

**Fig. 7:**
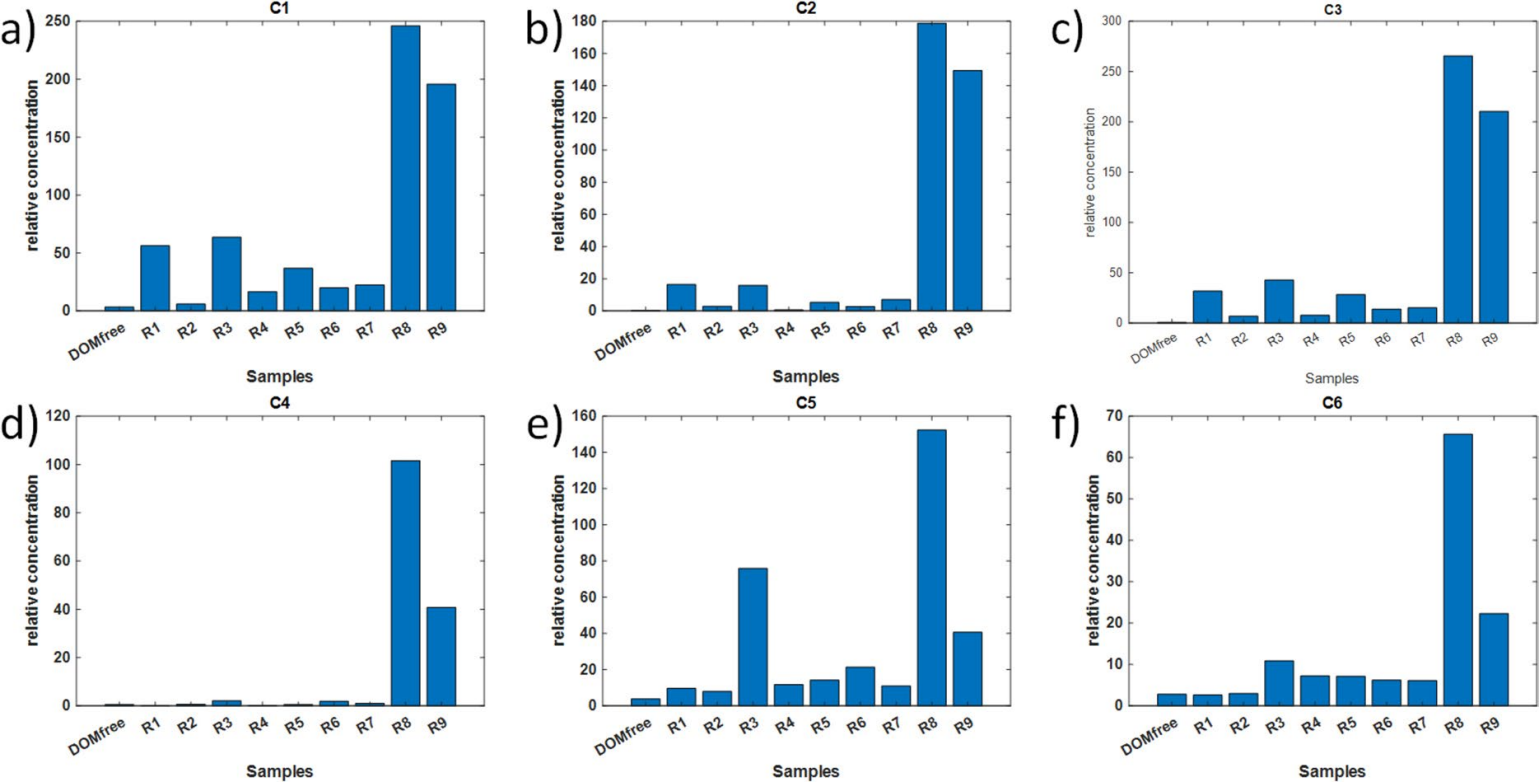
Relative contributions of each component in the ten water samples for the six MCR-ALS-resolved DOM components (a: C1, b: C2, c: C3, d: C4, e: C5, f: C6), obtained from the analysis of the augmented fluorescence EEM dataset

Humic-like fractions (C1, C2 and C3 in Fig. 6a-c and Table 2) dominated the DOM composition in low-altitude rivers, particularly in R8 and R9, which presented the highest contributions of all three components (Fig. 7a-c, and Supplementary Table 2). C1 (39% variance) were associated with terrestrial, recalcitrant DOM (Stedmon et al., 2003), while C2 (32% variance) and C3 (47.5% variance) represented smaller, less hydrophobic humic-fulvic substances indicative of anthropogenic influence (Murphy et al., 2011; Stedmon & Markager, 2005). These patterns aligned closely with the EIS PCA model results, where R9 was distinctly separated from the other samples, highlighting DOM enrichment due to urban and agricultural inputs.

The protein-like fractions (C4, C5 and C6 in Fig. 6 d-f and Table 2) were also most prominent in low-altitude rivers, suggesting significant anthropogenic and microbial contributions (see Fig. 7d-f, and Supplementary Table 2). C4 (9%) and C5 (16%) corresponded to tryptophan-like DOM (Mayer et al., 1999; Yamashita & Tanoue, 2003), indicative of anthropogenic contamination, with C5 being notably elevated in R3, a mid-altitude river. C6 (8%) represented a tyrosine-like autochthonous protein fraction (Coble, 1996), found across all rivers but reaching its highest levels in R8 and R9, reflecting elevated microbial activity. These results further corroborated the EIS PCA model, where low-frequency regions indicated higher DOM loads in R9, while mid- and high-altitude rivers presented intermediate or lower DOM loads.

For more details on the identification of MCR-ALS resolved fluorescence fractions, please see Supplementary Material.

### Assessment of EIS technique for DOM characterization

While individual analytical techniques provide valuable insights into different aspects of DOM composition, a single method alone is often insufficient to capture its full complexity. To achieve a more holistic understanding, EIS-derived parameters were integrated with classical physicochemical and spectroscopic data through a global PCA on the aggregated dataset, which included water temperature, pH, conductivity, fluorescence indices, MCR-ALS resolved DOM fractions, and the averaged PCA scores from the EIS model. This data combination approach integrated the complementary information provided by traditional techniques with EIS-derived data, enabling a comprehensive assessment of DOM variability across the samples. The correlation between all parameters simultaneously helped explain the distinct separation observed in low-altitude river water samples, such as R9, from the others, reinforcing the role of EIS as a reliable technique for DOM profiling.

The new PCA model, applied to the multi-source fused dataset, explained a total of 85% of the variance considering two principal components (PC1: 63%, PC2: 22%).

**Fig. 8:**
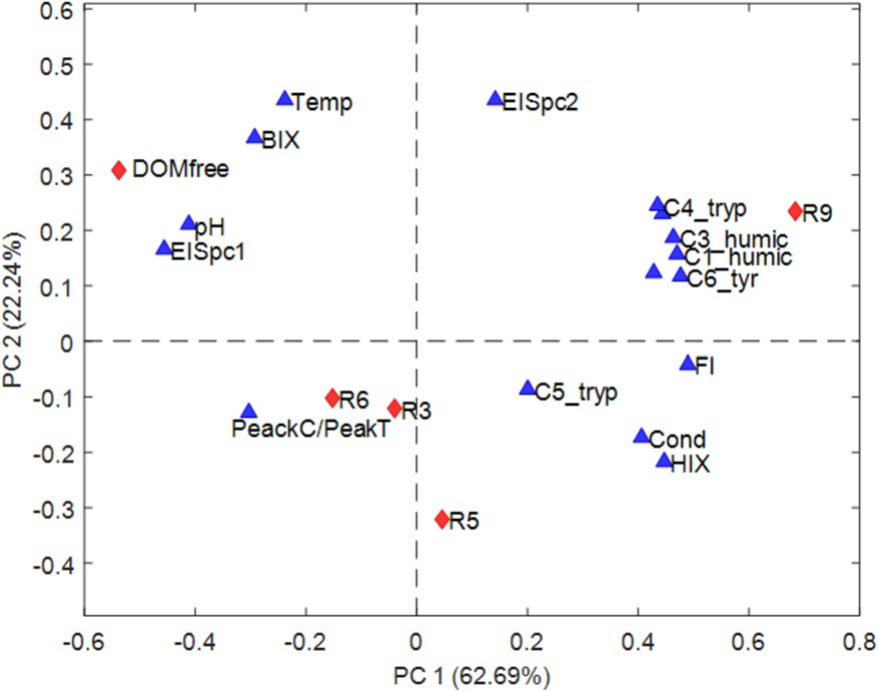
PCA results of the aggregated dataset. A PC1 vs. PC2 biplot illustrating the water samples scores (red diamonds) and the loadings (blue triangles), which represent water temperature, pH, conductivity, UV254, fluorescence indices, DOM fractions relative concentrations, and EIS scores

PC1 (Fig. 8) clearly distinguished R9 (red diamond) with the largest positive score, indicating a strong correlation with all DOM fractions, particularly humic-like and protein-like component which also showed large positive scores. This suggested a significant DOM presence of different characteristics in R9. Moreover, EISpc2 was positively correlated with R9 and all DOM fractions, as well as fluorescence indices (FI and HIX), high conductivity and low pH (negative loadings), pointing out its correlation with a high DOM load. These results suggested that the large phase angles observed at extremely low- and high-frequencies can be associated with substantial DOM content. In contrast, the DOM-free water had a negative PC1 score and clustered with pH, BIX and EISpc1, reflecting its lack of DOM and distinct electrochemical and optical characteristics. The corresponding large phase angles observed at middle to high frequencies further highlighted the distinct electrochemical behavior of this sample compared to DOM-containing samples. On PC2, EISpc1 and EISpc2 had positive loadings, contributing to the separation of DOM-free and R9 water samples from the other water samples.

R3, R5 and R6 clustered near PeakC/PeakT**,** suggesting a moderate presence of recalcitrant humic substances (Coble, 1996), whilst R9 strongly correlated with protein-like fractions and FI, indicating a higher presence of freshly produced, microbially influenced DOM (McKnight et al., 2001).

This integrated analysis confirmed that R9 behaved as the most distinct DOM profile, characterized by both humic and protein-like components, while DOM-free served as a control. These results highlighted the potential of EIS as a complementary technique for DOM characterization, as it allowed the differentiation between water samples with low and high DOM content and provided additional insight into DOM composition and variability.

## Conclusions

This study demonstrated that integrating EIS with traditional instrumental techniques, including UV–Vis molecular absorption and fluorescence spectroscopies, is an effective approach for characterizing DOM in river water samples. This multi-technique framework provided a more comprehensive understanding of DOM composition and distribution, proving EIS as a complementary tool to conventional optical methods.

The data aggregation strategy based on PCA confirmed and strengthened the results from individual techniques. The simultaneous analysis revealed clear distinctions in DOM distribution across river water samples and identified correlations between different EIS spectral regions and classical DOM indicators, such as UV254 absorbance, humic- and protein-associated indices and MCR-ALS resolved fractions. These relationships confirmed the robustness of the obtained results and enhanced the interpretability of EIS data, demonstrating its ability to capture key chemical characteristics of DOM in diverse aquatic environments.

Moreover, PCA of the EIS spectra established associations between phase angles at different frequency ranges and DOM load. Large phase angles at extremely low frequencies were strongly linked to high DOM content, while large phase angles at middle to high frequencies corresponded to lower DOM loads. These results suggest that EIS can serve as an effective, electrochemically driven indicator of DOM presence and variability, offering a novel perspective for rapid water quality assessment.

A strong altitudinal gradient was observed in DOM content, with low-altitude rivers exhibiting the highest DOM loads, followed by mid-altitude rivers, while high-altitude rivers contained the lowest DOM concentrations. This pattern aligns with expectations, as low-altitude rivers are more exposed to anthropogenic influences, whereas high-altitude rivers typically flow through pristine, minimally impacted environments.

## Supplementary Information

Below is the link to the electronic supplementary material.

ESM 1 Six MCR-ALS-resolved DOM components (**a**: C1, **b**: C2, **c**: C3, **d**: C4, **e**: C5, **f**: C6) obtained from the analysis of the augmented fluorescence EEM dataset: resolved excitation (blue) and emission (green) spectra. Average EEMs were calculated from duplicate measurements of a single representative sample per water type. (JPG 452 KB)

ESM 2 (DOCX 30.4 KB).

## Data Availability

Data has been uploaded to Zenodo repository. [Coupling Electrochemical and Spectroscopic Methods for River Water DOM Characterization](https://zenodo.org/records/16570763) : https://zenodo.org/records/16570763.

## References

[CR1] Aguilar-Torrejón, J. A., Balderas-Hernández, P., Roa-Morales, G., Barrera-Díaz, C. E., Rodríguez-Torres, I., & Torres-Blancas, T. (2023). Relationship, importance, and development of analytical techniques: COD, BOD, and TOC in water—an overview through time. *SN Applied Sciences,**5*, 118. 10.1007/s42452-023-05318-7

[CR2] Andersson, A., Lavonen, E., Harir, M., Gonsior, M., Hertkorn, N., Schmitt-Kopplin, Ph., Kylin, H., & Bastviken, D. (2020). Selective removal of natural organic matter during drinking water production changes the composition of disinfection by-products. *Environmental Science: Water Research & TechnoloGy,**6*, 779–794. 10.1039/C9EW00931

[CR3] Bonanos, N., Steele, B. C. H., Butler, E. P. Macdonald, J. R., Johnson, W. B., Worrell, W. L., Niklasson, G. A., Malmgren, S., Strømme, M., Sundaram, S. K., McKubre, M., Macdonald, D., Engelhardt, G., Barsoukov, E., Conway, B., Pell,W., Wagner, N., Roland, C., & Eisenberg, R. (2018). Applications in Impedance Spectroscopy. In E. Barsoukov, & R. MacDonald (Eds.), *Impedance Spectroscopy* (pp 175−478). Wiley Online Books, John Wiley & Sons.

[CR4] Buchicchio, E., De Angelis, A., Santoni, F., Carbone, P., Bianconi, F., & Smeraldi, F. (2023). Battery SOC estimation from EIS data based on machine learning and equivalent circuit model. *Energy,**283*, 128461. 10.1016/j.energy.2023.128461

[CR5] Carstea, E., Popa, C., Baker, A., & Bridgeman, J. (2020). In situ fluorescence measurements of dissolved organic matter: A review. *Science of the Total Environment,**669*, 134361. 10.1016/j.scitotenv.2019.134361 10.1016/j.scitotenv.2019.134361 31683216

[CR6] Coble, A. A., Wymore, A. S., Potter, J. D., & McDowell, W. H. (2022). Land use overrides stream order and season in driving dissolved organic matter dynamics throughout the year in a river network. *Environmental Science & Technology,**56*, 2009–2020. 10.1021/acs.est.1c06305 35007420 10.1021/acs.est.1c06305

[CR7] Coble, P. G. (1996). Characterization of marine and terrestrial DOM in seawater using excitation-emission matrix spectroscopy. *Marine Chemistry,**51*, 325–346. 10.1016/0304-4203(95)00062-3

[CR8] de Juan, A., & Tauler, R. (2001). Comparison of three-way resolution methods for non-trilinear chemical data sets. *Journal of Chemometrics,**15*, 749–771. 10.1002/cem.662

[CR9] Dean, J. F., & Battin, T. J. (2024). Future directions for river carbon biogeochemistry observations. *Nature Water,**2*, 219–222. 10.1038/s44221-024-00207-8

[CR10] Ding, H., Zheng, M., Yan, L., Zhang, X., Liu, L., Sun, Y., Su, J., Xi, B., & Yu, H. (2025). Spectral and molecular insights into the variations of dissolved organic matter in shallow groundwater impacted by surface water recharge. *Water Research,**273*, 122978. 10.1016/j.watres.2024.122978 39765096 10.1016/j.watres.2024.122978

[CR11] Du, H., Chen, G., & Wang, J. (2023). Highly selective electrochemical impedance spectroscopy-based graphene electrode for rapid detection of microplastics. *Science of Total Environment,**862*, 160873. 10.1016/j.scitotenv.2022.160873 10.1016/j.scitotenv.2022.160873 36521612

[CR12] Fellman, J., Hood, E., & Spencer, G. (2010). Fluorescence spectroscopy opens new windows into dissolved organic matter dynamics in freshwater ecosystems: A review. *Limnology and Oceanography,**55*, 2452–2462. 10.4319/lo.2010.55.6.2452

[CR13] Gad, M., Khomami, N., Krieg, R., Schor, J., Philippe, A., & Lechtenfeld, O. (2025). Environmental drivers of dissolved organic matter composition across Central European aquatic systems: A novel correlation-based machine learning and FT-ICR MS approach. *Water Research,**273*, 123018. 10.1016/j.watres.2024.123018 39742633 10.1016/j.watres.2024.123018

[CR14] Gan, L., Yan, Z., Ma, Y., Zhu, Y., Li, X., Xu, J., & Zhang, W. (2019). pH dependence of the binding interactions between humic acids and bisphenol A - A thermodynamic perspective. *Environmental Pollution,**255*, 113292. 10.1016/j.envpol.2019.113292 31597112 10.1016/j.envpol.2019.113292

[CR15] Gao, R., Wang, H., Abdurahman, A., Liang, W., Lu, X., Wei, S., & Zeng, F. (2022). Insight into the hetero-interactions of 4-nonylphenol with dissolved organic matter: Multiple spectroscopic methods, ^1^H NMR study and principal component analysis. *RSC Advances,**12*, 22416–22424. 10.1039/d2ra03739d 36105990 10.1039/d2ra03739d PMC9364969

[CR16] Gold, A., Thompson, S. P., & Piehler, M. F. (2019). The effects of urbanization and retention-based stormwater management on coastal plain stream nutrient export. *Water Resources Research,**55*, 7027–7046. 10.1029/2019wr024769

[CR17] Huguet, A., Vacher, L., Relexans, S., Saubusse, S., Froidefond, J. M., & Parlanti, E. (2009). Properties of fluorescent dissolved organic matter in the Gironde Estuary. *Organic Geochemistry,**40*, 706–719. 10.1016/j.orggeochem.2009.03.002

[CR18] Jaffé, R., McKnight, D., Maie, N., Cory, R., McDowell, W. H., & Campbell, J. (2008). Spatial and temporal variations in DOM composition in ecosystems: The importance of long-term monitoring of optical properties. *Journal of Geophysical Research,**113*, G04032. 10.1029/2008JG000683

[CR19] Jansen, B., Kalbitz, K., & McDowell, W. (2014). Dissolved organic matter: Linking soils and aquatic systems. *Vadose Zone Journal,**13*(7), 1–4. 10.2136/vzj2014.05.0051

[CR20] Jaumot, J., de Juan, A., & Tauler, R. (2015). MCR-ALS GUI 2.0: New features and applications. *Chemometrics and Intelligent Laboratory Systems,**140*, 1–12. 10.1016/j.chemolab.2014.10.003

[CR21] Jolliffe, I. (2011). Principal Component Analysis. In M. Lovric (Ed.), *International Encyclopedia of Statistical Science* (pp. 1094–1096). Springer. 10.1007/978-3-642-04898-2_455

[CR22] Kellerman, A., Dittmar, T., Kothawala, D., & Tranvil, L. (2014). Chemodiversity of dissolved organic matter in lakes driven by climate and hydrology. *Nature Communications,**5*, 3804. 10.1038/ncomms4804 10.1038/ncomms4804 24787272

[CR23] Kowalczuk, P., Stoń-Egiert, J., Cooper, W. J., Whitehead, R., & Durako, M. (2005). Characterization of chromophoric dissolved organic matter (CDOM) in the Baltic Sea by excitation emission matrix fluorescence spectroscopy. *Marine Chemistry,**96*, 273–292. 10.1016/j.marchem.2005.03.002

[CR24] Lawal, A. T. (2023). Recent developments in electrochemical sensors based on graphene for bioanalytical applications. *Sensing and Bio-Sensing Research,**41*, 100571. 10.1016/j.sbsr.2023.100571

[CR25] Li, P., & Hur, J. (2017). Utilization of UV-Vis spectroscopy and related data analyses for dissolved organic matter (DOM) studies: A review. *Critical Reviews in Environmental Science and Technology,**47*, 131–154. 10.1080/10643389.2017.1309186

[CR26] Ludwig, G. M., Hobbs, M. S., & Perschbacher, P. W. (2007). Ammonia, pH, and plankton in sunshine bass nursery ponds: The effect of inorganic fertilizer or sodium bicarbonate. *North American Journal of Aquaculture,**69*, 80–89. 10.1577/a05-078.1

[CR27] Lvovich, V. F. (2014). Electrochemical Impedance Spectroscopy (EIS) Applications to Sensors and Diagnostics. In: G. Kreysa, Ki. Ota, R. F. Savinell (Eds.), *Encyclopedia of Applied Electrochemistry*. Springer, New York, NY. 10.1007/978-1-4419-6996-5_67

[CR28] Marcé, R., Verdura, L., & Leung, N. (2021). Dissolved organic matter spectroscopy reveals a hot spot of organic matter changes at the river–reservoir boundary. *Aquatic Science,**83*, 67. 10.1007/s00027-021-00823-6

[CR29] Marchetto, A., Mosello, R., Psenner, R., Bendetta, G., Boggero, A., Tait, D., & Tartari, G. (1995). Factors affecting water chemistry of alpine lakes. *Aquatic Sciences,**57*, 81–89. 10.1007/BF00878028

[CR30] Marín-García, M., & Tauler, R. (2020). Chemometrics characterization of the Llobregat River dissolved organic matter. *Chemometrics and Intelligent Laboratory Systems,**201*, 104018. 10.1016/j.chemolab.2020.104018

[CR31] Mayer, L. M., Schick, L. L., & Loder, T. C., III. (1999). Dissolved protein fluorescence in two Maine estuaries. *Marine Chemistry,**64*, 171–179. 10.1016/S0304-4203(98)00072-3

[CR32] McKnight, D. M., Boyer, E. W., Westerhoff, P. K., Doran, P. T., Kulbe, T., & Andersen, D. T. (2001). Spectrofluorometric characterization of dissolved organic matter for indication of precursor organic material and aromaticity. *Limnology and Oceanography,**46*, 38–48. 10.4319/lo.2001.46.1.0038

[CR33] Mitschke, N., Vemulapalli, S. P. B., & Dittmar, T. (2023). NMR spectroscopy of dissolved organic matter: A review. *Environmental Chemistry LettErs,**21*, 689–723. 10.1007/s10311-022-01528-4

[CR34] Murphy, K. R., Stedmon, C. A., Graeber, D., & Bro, R. (2013). Fluorescence spectroscopy and multi-way techniques. PARAFAC. *Analytical Methods,**5*, 6557–6566. 10.1039/C3AY41160E

[CR35] Murphy, K., Hambly, A., Singh, S., Henderson, R., Baker, A., Stuetz, R., & Khan, S. (2011). Toward a unified PARAFAC model. *Environmental Science & Technology,**45*, 2909–2916. 10.1021/es103015e 21361278 10.1021/es103015e

[CR36] Murphy, K., Stedmon, C., Waite, T. D., & Ruiz, G. (2008). Distinguishing between terrestrial and autochthonous organic matter sources in marine environments using fluorescence spectroscopy. *Marine Chemistry,**108*, 40–58. 10.1016/j.marchem.2007.10.003

[CR37] Ohno, T. (2002). Fluorescence inner-filtering correction for determining the humification index of dissolved organic matter. *Environmental Science & Technology,**36*, 742–746. 10.1021/es0155276 11878392 10.1021/es0155276

[CR38] Patrone, J., Vila-Costa, M., Dachs, J., Papazian, S., Gago-Ferrero, P., & Gil-Solsona, R. (2024). Enhancing molecular characterization of dissolved organic matter by integrative direct infusion and liquid chromatography nontargeted workflows. *Environmental Science & Technology,**58*, 12454–12466. 10.1021/acs.est.4c00876 38958378 10.1021/acs.est.4c00876 PMC11256763

[CR39] Ren, Y., Liu, S., Liu, L., Suo, C., Fu, R., Zhang, Y., Qiu, Y., & Wu, F. (2024). Deciphering the molecular composition and sources of dissolved organic matter in urban rivers based on optical spectroscopy and FT-ICR-MS analyses. *Carbon Research,**3*, 67. 10.1007/s44246-024-00151-y

[CR40] Serrano-Pallicer, E., Muñoz-Albero, M., Pérez-Fuster, C., Peris, R., & Laguarda-Miró, N. (2018). Early detection of freeze damage in navelate oranges with electrochemical impedance spectroscopy. *Sensors,**18*(12), 4503. 10.3390/s18124503 30572655 10.3390/s18124503 PMC6308850

[CR41] Song, X., Zhao, M., Chen, A., Xie, X., Yang, H., Zhang, S., Wei, Z., & Zhao, Y. (2022). Effects of input of terrestrial materials on photodegradation and biodegradation of DOM in rivers: The case of Heilongjiang River. *Journal of Hydrology,**609*, 127792. 10.1016/j.jhydrol.2022.127792

[CR42] Stedmon, C. A., & Markager, S. (2005). Tracing the production and degradation of autochthonous fractions of dissolved organic matter by fluorescence analysis. *Limnology and Oceanography,**50*(5), 1415–1426.

[CR43] Stedmon, C. A., Markager, S., & Bro, R. (2003). Tracing dissolved organic matter in aquatic environments using a new approach to fluorescence spectroscopy. *Marine Chemistry,**82*(3–4), 239–254. 10.1016/S0304-4203(03)00072-0

[CR44] Strobel, B. W., Borggaard, O. K., Hansen, H. C. B., Andersen, M., & Raulund-Rasmussen, K. (2004). Dissolved organic carbon and decreasing ph mobilize cadmium and copper in soil. *European Journal of Soil Science,**56*, 189–196. 10.1111/j.1365-2389.2004.00661.x

[CR45] Sung, W. (2011). Technical note: Using UV-vis spectrophotometry to estimate nitrite plus nitrate and monochloramine. *Journal of the American Water Works Association,**103*, 97–103. 10.1002/j.1551-8833.2011.tb11476.x

[CR46] Tauler, R. (1995). Multivariate curve resolution applied to second order data. *Chemometrics and Intelligent Laboratory Systems,**30*, 133–146. 10.1016/0169-7439(95)00047-X

[CR47] Tauler, R. (2020). Multivariate curve resolution of multiway data using the multilinearity constraint. *Journal of Chemometrics,**35*, e3279. 10.1002/cem.3279

[CR48] Tauler, R., Marqués, I., & Casassas, E. (1998). Multivariate curve resolution applied to three-way trilinear data: Study of a spectrofluorimetric acid-base titration of salicylic acid at three excitation wavelengths. *Journal of Chemometrics,**12*, 55–75. 10.1002/(SICI)1099-128X(199801/02)12:13.0.CO;2-%23

[CR49] UNESCO. (2022). I*HP-IX: Strategic Plan of the Intergovernmental Hydrological Programme: Science for a Water Secure World in a Changing Environment, ninth phase 2022-2029. *Retrieved August 19, 2025, from https://unesdoc.unesco.org/ark:/48223/pf0000381318

[CR50] UN-Water. (2021). *Summary progress update 2021*: *SDG 6 – water and sanitation for all. *Retrieved August 19, 2025, from https://www.unwater.org/sites/default/files/app/uploads/2021/12/SDG-6-Summary-Progress-Update-2021_Version-July-2021a.pdf

[CR51] Vera, M., Cruz, S., Boleda, M. R., Mesa, J., Martín-Alonso, J., Casas, S., Gibert, O., & Cortina, J. L. (2017). Fluorescence spectroscopy and parallel factor analysis as a dissolved organic monitoring tool to assess treatment performance. *Science of Total Environment,**584–585*, 1212–1220. 10.1016/j.scitotenv.2017.01.184 10.1016/j.scitotenv.2017.01.184 28169026

[CR52] Weishaar, J., Aiken, G., Bergamaschi, B., Fram, M., Fujii, R., & Mopper, K. (2003). Evaluation of specific ultraviolet absorbance as an indicator of the chemical composition and reactivity of dissolved organic carbon. *Environmental Science & Technology,**37*, 4702–4708. 10.1021/es030360x 14594381 10.1021/es030360x

[CR53] Windig, W., & Stephenson, D. A. (1992). Self-modeling mixture analysis of second-derivative near-infrared spectral data using the simplisma approach. *Analytical Chemistry,**64*, 2735–2742. 10.1021/ac00046a015

[CR54] Yamashita, Y., & Tanoue, E. (2003). Chemical characterization of protein-like fluorophores in DOM in relation to aromatic amino acids. *Marine Chemistry, 82*, 255–271. 10.1016/S0304-4203(03)00073-2

[CR55] Yamashita, Y., & Jaffé, R. (2008). Characterizing the interactions between trace metals and dissolved organic matter using excitation−emission matrix and parallel factor analysis. *Environmental Science & Technology,**42*, 7374–7379. 10.1021/es801357h 18939573 10.1021/es801357h

[CR56] Zepp, R. G., Sheldon, W. M., & Moran, M. A. (2004). Dissolved organic fluorophores in southeastern US coastal waters: Correction method for eliminating Rayleigh and Raman scattering peaks in excitation–emission matrices. *Marine Chemistry,**89*, 15–36.

[CR57] Zhang, X., Marcé, R., Armengol, J., & Tauler, R. (2014). Distribution of dissolved organic matter in freshwaters using excitation emission fluorescence and multivariate curve resolution. *Chemosphere,**111*, 120–128. 10.1016/j.chemosphere.2014.03.009 24997908 10.1016/j.chemosphere.2014.03.009

[CR58] Zhang, Y., et al. (2009). Characterization of dissolved organic matter in urban stormwater runoff by UV–Vis absorbance and fluorescence spectroscopy. *Water Research,**43*, 2498–2506.

[CR59] Zhang, Y., van Dijk, M. A., Liu, M., Zhu, G., & Qin, B. (2009). The contribution of phytoplankton degradation to chromophoric dissolved organic matter (DOM) in eutrophic shallow lakes: Field and experimental evidence. *Water Research,**43*, 4685–4697. 19665748 10.1016/j.watres.2009.07.024

