# Supplementary figures and images for: Coupling electrochemical and spectroscopic methods for river water dissolved organic matter characterization
Source: Environ Monit Assess. 2025 Sep 1;197(9):1071. doi: 10.1007/s10661-025-14489-2 (PMC12402017; doi:10.1007/s10661-025-14489-2)

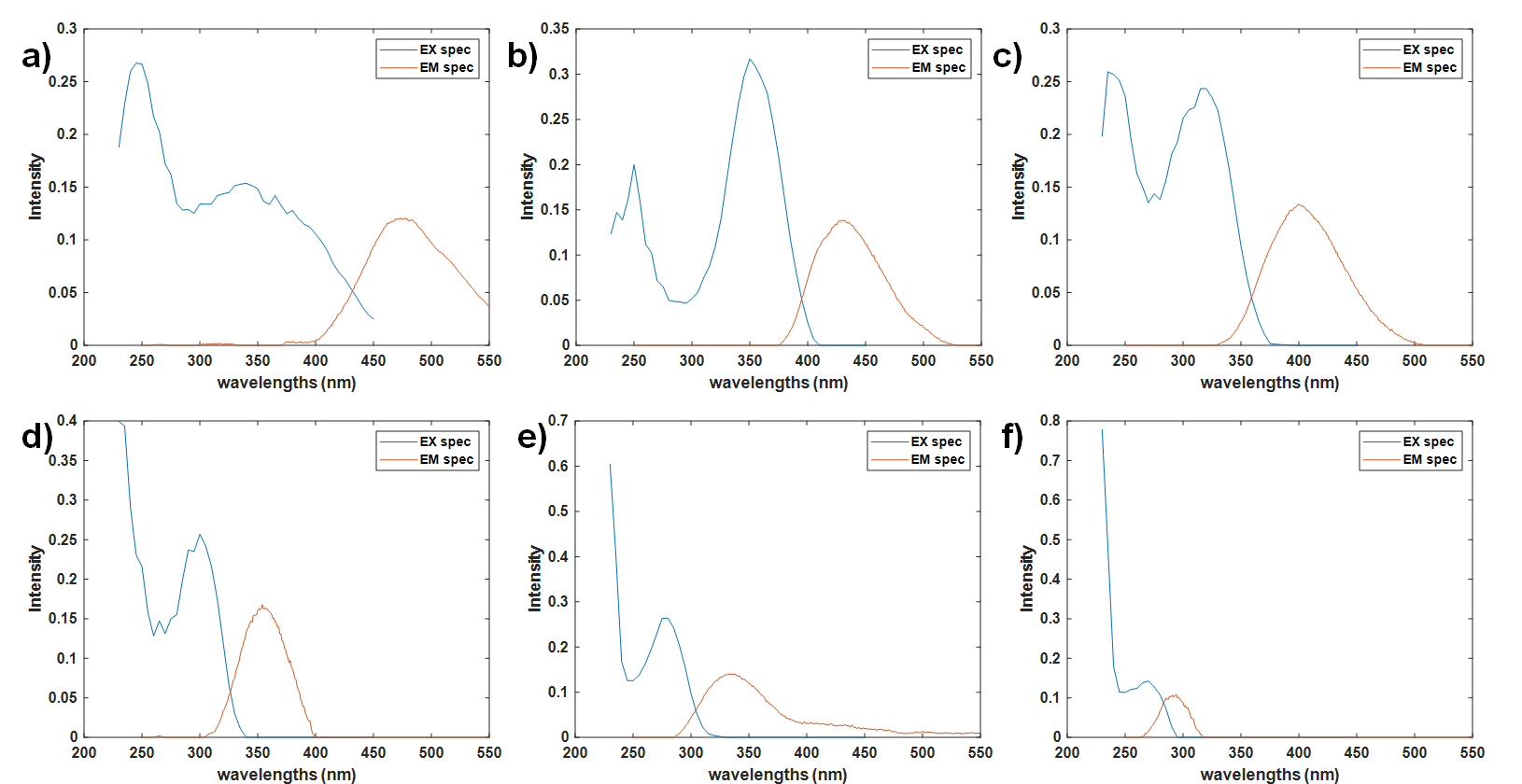

Supplement: Supplementary file 1 — Six MCR-ALS-resolved DOM components (a: C1, b: C2, c: C3, d: C4, e: C5, f: C6) obtained from the analysis of the augmented fluorescence EEM dataset: resolved excitation (blue) and emission (green) spectra. Average EEMs were calculated from duplicate measurements of a single representative sample per water type. (JPG 452 KB) [file 10661_2025_14489_MOESM1_ESM.jpg]
