# Supplementary material for: Coupling electrochemical and spectroscopic methods for river water dissolved organic matter characterization
Source: Environ Monit Assess. 2025 Sep 1;197(9):1071. doi: 10.1007/s10661-025-14489-2 (PMC12402017; doi:10.1007/s10661-025-14489-2)
Supplement: Supplementary file 2 — (DOCX 30.4 KB). [file 10661_2025_14489_MOESM2_ESM.docx]

**Supplementary information**

**Material and methods**

Sampling

**Supplementary Table1.** River samples characteristics and their potential environmental impacts

| Code | Rivers and  sample locations | Altitude | Environmental impact | T °C | pH | Conductivity µS |
| --- | --- | --- | --- | --- | --- | --- |
| R1 | Angostrina 42°29'23.1"N 1°57'45.3"E | High  1260m asl | Mixed natural and agricultural influences, active tourism | 9.8 | 6.7 | 29.7 |
| R2 | Font Freda 42°20'53.7"N 1°51'11.0"E | High  1280m asl | Minimal human impact, pristine environment | 10.1 | 7.0 | 423 |
| R3 | Segre 42°23'20.1"N 1°50'50.4"E | Middle  1000m asl | Mixed natural and agricultural influences  active tourism | 12.4 | 7.4 | 143 |
| R4 | Riu de la Vila 42°23'12.8"N 2°02'06.3"E | High  1470m asl | Minimal human impact, pristine environment | 8.5 | 7.4 | 72.6 |
| R5 | Riu D’Alp 42°20'38.1"N 1°57'28.1"E | High  1600m asl | Minimal human impact, pristine environment | 8.2 | 7.3 | 567 |
| R6 | Freser 42°15'46.5"N,2°09'42.2"E | Middle  880m asl | Mixed natural and agricultural influences, active tourism | 12 | 7.6 | 348 |
| R7 | Cassibros 42°34'19.5"N 1°14'02.5"E | Middle  1000m asl | Minimal human impact, pristine environment | 8.7 | 7.8 | 64.6 |
| R8 | Ter  41°58'4.92"N  2°18'48.67"E | Low  500m asl | Urban activities, agriculture, urban runoff | 14.2 | 7.1 | 1625 |
| R9 | Ter 41°58'47.81" N2°19'37.96" E | Low  480m asl | Urban activities, agriculture, wastewater treatment | 14.5 | 7.3 | 594 |
| DOM-free | Milli-Q water |  |  | 24.3 | 7.7 | 1.7 |

* High-altitude river water samples are defined as those collected above 1200m asl; mid-altitude water samples are those collected between 800 and 1200m asl; and low-altitude river water samples are those collected below 800m asl.

The high-altitude rivers, located in the alpine Catalan Pyrenees region, included the Angostrina (R1), Font Freda (R2), Riu de la Vila (R4), and Riu D’Alp (R5). These rivers are situated in mountainous areas with minimal human impact, providing insights into DOM composition in pristine, natural environments.

The mid-altitude Catalan rivers included the Segre (R3), Freser (R6), and Cassibros (R7). The Segre River, a major tributary of the Ebro River, flows through both Catalonia and Aragon, passing through touristic areas with moderate agricultural activity. The Angostrina River flows through the Pyrenean region, influenced by active tourism and agricultural practices. Among these, the Cassibros River (R7) is the most pristine, experiencing minimal human impact.

Low-altitude samples were collected from two sites on the Ter River: one upstream (R8) and one downstream of a wastewater treatment plant (R9). The Ter River flows through central Catalonia, passing through several urban areas and agricultural zones, both of which are expected to impact its DOM content.

Fluorescence indices

**Fluorescence index (FI).** This index is primarily used to identify the sources of DOM. FI is calculated as the ratio of fluorescence emission intensities at wavelengths 470 nm and 520 nm, using an excitation wavelength of 370 nm (Cory and McKnight, 2005).

FI = I_em470_/I_em520_, at Ex = 370 nm **Equation 1**

**Humification index (HIX).** This index reflects the degree of humic content and DOM maturation. HIX was computed as the area under the emission curve from wavelength 435 to 480 nm divided by the area from 300 to 345 nm, using an excitation wavelength of 255 nm (Ohno, 2002).

HIX = Σ I_Em435-480_ / Σ I_Em300-345_, at Ex = 254 nm **Equation 2**

**Biological index (BIX).** This index serves as an indicator of the autochthonous biological production of organic matter. BIX was calculated as the ratio of emission intensities at wavelength 380 nm and 430 nm, using an excitation wavelength of 310 nm (Huguet et al., 2009).

BIX = I_em380_/I_em430_, at Ex = 310 nm **Equation 3**

**Peak C/Peak T ratio.** This ratio provides insights into the balance between humic-like (recalcitrant) and protein-like (labile) dissolved organic matter (DOM) in a water sample (Coble, 1996). Peak C corresponds to humic substances, which are typically more stable, aromatic, and resistant to degradation. In contrast, Peak T represents protein-like, freshly produced organic matter that is more biologically available and rapidly decomposed. Peak C and Peak T are calculated based on fluorescence intensities. Peak C corresponds to the maximum emission intensity within the range of 420–480 nm at an excitation wavelength of 350 nm, while Peak T corresponds to the maximum emission intensity within the range 330 -350 nm at an excitation wavelength of 275 nm.

PeakC /PeakT = maxI_ex350,em420-480_ / maxI_ex275,em330-350_ **Equation 4**

The values calculated for each index provide unique insights into the DOM characteristics. However, these indices are not universally applicable, as various factors, including environmental conditions and experimental settings (e.g., water type, seasonal temperature variations and others), can influence their values and subsequent interpretation (Huguet et al., 2009).

**Results**

Fluorescence indices

The FI values ranged from 1.3 to 2.8 across the samples, indicating a variation between terrestrial and microbial DOM sources. Generally, FI values below 1.4 suggest a predominantly terrestrial source, while values above 1.9 imply a microbial origin (McKnight et al., 2001). In this study, samples R2 and R4, with FI values of 1.30 and 1.43, respectively, were likely influenced by terrestrial sources. Conversely, samples with higher FI values, such as R5, R7, R8, and R9 (1.95, 1.99, 2.72 and 2.78, respectively), suggested a stronger microbial influence. The highest FI values, observed in R8 and R9, collected before and after a wastewater treatment plant (WTP) on the Ter River, indicated a significant contribution of microbial derived DOM, likely impacted by urban and agricultural inputs.

The **HIX** values across all samples were below 1.0, indicating a low degree of humification and suggesting the presence of freshly produced or autochthonous organic matter. Typically, HIX values between 10 and 16 denote a strong humic character, primarily from terrestrial sources (Ohno, 2002), whereas values below 4 reflect less humified, autochthonous material. In this study, the consistently low HIX values (0.49 to 0.92) indicated DOM of recent microbial or autochthonous origin. This interpretation was particularly plausible given that the sampling campaign took place in spring, following the cold winter temperatures in the Catalan Pyrenees. As temperatures rise, microbial activity tends to increase, leading to the production of recently generated DOM. This seasonal factor supported the observation of low HIX values, as microbial communities become more active and contributed with freshly derived DOM to the river waters.

The BIX values ranged from 0.5 to 0.9, with most samples presenting values below 0.8. A BIX value above 1.0 typically indicates high autochthonous biological activity, while values between 0.6 and 0.7 suggest lower DOM production in natural waters (Huguet et al., 2009). In this study, only sample R8 presented a BIX value approaching 0.9, indicating moderate biological activity and the presence of freshly produced DOM. The generally low BIX values across the samples reflected stable DOM conditions with reduced biodegradation impacts. This observed stability was consistent with the alpine samples (R2, R4, R5, R6, and R7), where natural waters are less affected by anthropogenic impacts and microbial degradation compared to lower-altitude sites.

The **Peak C/Peak T ratios** further supported the results on DOM composition. Higher ratios in **R1, R5, R8, and R9** (3.1, 1.6, 1.5, and 2.9, respectively) suggested a dominance of **recalcitrant, humic-like DOM,** likely due to the accumulation of humic substances. The higher ratios in the high-altitude rivers **R1 and R5** may result from colder spring temperatures, which limited microbial degradation and allowed humic DOM to persist. In contrast, the increase in the ratio from **R8 (upstream of the WTP) to R9 (downstream of the WTP)** suggested that the treatment process likely removed much of the protein fractions, shifting the balance toward humic fractions. Lower ratios in mid-altitude rivers **R3 and R6** indicated a higher proportion of **protein-like, freshly produced DOM. This fact was** likely due to recently activated microbial activity or anthropogenic inputs, such as agricultural runoff.

The generally low HIX values, combined with moderate FI and low BIX, suggested that the DOM composition across these rivers is relatively fresh, with a mix of autochthonous and anthropogenic influences rather than highly humified, terrestrial DOM. Particularly in the Ter samples, the elevated FI and moderate BIX may indicate some urban and agricultural contributions, resulting in a higher fraction of microbially derived DOM. In contrast, the alpine rivers (e.g., R2, R4) presented FI and HIX values aligned with the expected lower impact of human activity, reflecting a more pristine DOM composition mainly of recent microbial origin.

MCR-ALS DOM fractions identification

**Supplementary Fig. 1 near here**

The first component, **C1** (humic-like fraction), in main text Fig.6a and in the Supplementary Fig. 1a, accounted for 39% of the DOM in the river water samples. According to the literature, these spectral characteristics are indicative of high molecular weight, hydrophobic terrestrial humic substances (Coble, 1996; Stedmon et al., 2003; Baghoth et al., 2011). The highest concentrations of this component were found in samples R8 and R9. Samples R1 and R3 from the mid-altitude rivers also showed notable levels (see Figure 7), while it was almost absent in the DOM free water.

As shown in Fig. 6b, the second component (**C2**) accounted for 32% of the data variance. This component presented excitation maxima at <260 nm and 350 nm, with an emission maximum at 430 nm (and Supplementary Fig. 1b). These spectral features are commonly associated with terrestrial and/or anthropogenic humic-fulvic-like DOM, characterized by smaller and less hydrophobic molecules than heavier humic substances (Coble, 1996; Stedmon & Markager, 2005). This type of DOM was primarily observed in samples R8 and R9 in Fig. 7b, while it was barely detectable in the high-altitude mountain samples. Small concentrations were also present in mid-altitude river samples, R1 and R3.

In Figure 6c, the third MCR-ALS component (**C3**) accounted for the largest portion of data variance at 47.5%, compared to the other components. This component showed two distinct excitation maxima: one below 250 nm and another at 320 nm, both associated with a common emission maximum at 399 nm (and Supplementary Fig. 1c). According to the literature, these maxima are characteristic of terrestrial-like humic substances with relatively low molecular weights, potentially indicating recent microbial activity driven by rising spring temperatures (Coble, 1996; Zhang et al., 2009; Murphy et al., 2011). This DOM fraction DOM was observed across all river water samples in this study, with the highest percentages found in samples R8 and R9 (Fig. 7c). Additionally, samples R1 and R3, collected from mid-altitude Catalan rivers, showed notable concentrations of this component compared to other mountain rivers. This fact was likely due to advanced spring temperatures enhancing biological activity within these rivers.

In Fig. 6d-f, three components (**C4**, **C5**, and **C6**) were identified in the EEM regions corresponding to protein-like fluorophores (Coble, 1996; Mayer et al., 1999; Yamashita & Tanoue, 2003). The fourth component, **C4** in Fig. 6d, accounting for 9% of the variance, showed two excitation maxima at <240 nm and 300 nm, with an emission maximum at 354 nm (Supplementary Fig. 1d). In contrast, the **C5** peak was located at <240 and 280-nm excitation maxima, with an emission maximum at 338nm, as shown in Supplementary Fig. 1e. This fraction accounted for less than 16% across all water matrices. Based on the literature, both components can be attributed to protein-like fractions within DOM, with fluorescence characteristics similar to those produced by tryptophan. These protein-like fractions were predominantly found in R8 and R9, suggesting that water quality was significantly impacted by anthropogenic and agricultural activities in the low-altitude rivers (see Fig. 7e). Interestingly, **C5** was also prominent in R3, suggesting anthropogenic contamination.

Similarly, **C6** (8% explained variance) had excitation and emission characteristics similar to those of an autochthonous protein-like, tyrosine-like fraction. This component presented excitation maxima at <240nm and 270 nm, both associated with a common emission maximum at 294 nm (Supplementary Fig. 1f). Since this DOM fraction is typical for the autochthonous habitat, it was well distributed across all rivers. Obviously, it was in highest concentrations in both Ter rives samples, but **C6** was also found in the mountains at high altitudes (Fig. 7f).

| River Water Sample | Fraction 1 | Fraction 2 | Fraction 3 | Fraction 4 | Fraction 5 | Fraction 6 |
| --- | --- | --- | --- | --- | --- | --- |
| MilliQ | 3.447 | 0.318 | 0.707 | 0.548 | 3.837 | 2.807 |
| R1 | 56.256 | 16.400 | 31.775 | 0.000 | 9.638 | 2.624 |
| R2 | 5.982 | 2.869 | 6.916 | 0.674 | 7.946 | 2.994 |
| R3 | 63.648 | 15.912 | 42.849 | 2.073 | 75.887 | 10.893 |
| R4 | 16.561 | 0.603 | 7.744 | 0.120 | 11.716 | 7.233 |
| R5 | 36.803 | 5.292 | 28.413 | 0.511 | 14.216 | 7.128 |
| R6 | 19.903 | 2.747 | 13.892 | 1.861 | 21.332 | 6.236 |
| R7 | 22.441 | 7.047 | 15.297 | 0.978 | 11.014 | 6.107 |
| R8 | 246.136 | 178.767 | 265.636 | 101.585 | 152.366 | 65.630 |
| R9 | 195.792 | 149.368 | 210.486 | 40.773 | 40.679 | 22.275 |

**Supplementary Table 2,** Resolved MCR-ALS relative contributions (concentrations) for the six DOM fractions in the ten river water samples

**References**

Baghoth, S., Sharma, S., & Amy, G. (2011). Tracking natural organic matter (NOM) in a drinking water treatment plant using fluorescence excitation–emission matrices and PARAFAC. *Water Research, 45*, 797-809. https://doi.org/10.1016/j.watres.2010.09.005

Coble, P.G. (1996). Characterization of marine and terrestrial DOM in seawater using excitation-emission matrix spectroscopy. *Marine Chemistry, 51*, 325-346. <https://doi.org/10.1016/0304-4203(95)00062-3>

Cory, R.M., & McKnight, D.M. (2005). Fluorescence spectroscopy reveals ubiquitous presence of oxidized and reduced quinones in dissolved organic matter. *Environmental Science and Technology, 39*(21), 8142-8149. doi:10.1021/es0506962.

Mayer, L.M., Schick, L.L., & Loder III, T.C. (1999). Dissolved protein fluorescence in two Maine estuaries. *Marine Chemistry, 64,* 171–179. https://doi.org/10.1016/S0304-4203(98)00072-3

Murphy , K., Hambly, A., Singh, S., Henderson, R., Baker, A., Stuetz, R., & Khan, S. 2011. Toward a Unified PARAFAC Model. *Environmental Science and Technology, 45*, 2909-2916. <https://doi.org/10.1021/es103015e>

Ohno, T. (2002). Fluorescence inner-filtering correction for determining the humification index of dissolved organic matter. *Environmental Science and Technology, 36*, 742–746. https://doi. org/10.1021/es0155276.

Huguet, A., Vacher, L., Relexans, S., Saubusse, S., Froidefond, J.M., & Parlanti, E. (2009). Properties of fluorescent dissolved organic matter in the Gironde Estuary. *Organic Geochemistry, 40*, 706–719. <https://doi.org/10.1016/j.orggeochem.2009.03.002>

Stedmon, C.A., & Markager, S. (2005). Tracing the production and degradation of autochthonous fractions of dissolved organic matter by fluorescence analysis. *Limnology and Oceanography 50* (5), 1415-1426.

Stedmon, C.A., Markager, S., & Bro, R. (2003). Tracing dissolved organic matter in aquatic environments using a new approach to fluorescence spectroscopy. *Marine Chemistry, 82* (3-4), 239-254. https://doi.org/10.1016/S0304-4203(03)00072-0 Mar.

**Zhang, Y., et al. (2009).** Characterization of dissolved organic matter in urban stormwater runoff by UV–Vis absorbance and fluorescence spectroscopy. *Water Research*, **43,** 2498–2506.

Yamashita, Y., & Tanoue, E. (2003). Chemical characterization of protein-like fluorophores in DOM in relation to aromatic amino acids. Marine Chemistry, 82, 255-271. https://doi.org/10.1016/S0304-4203(03)00073-2
